# Supplementary material for: Cell type diversity in a developing octopus brain
Source: Nat Commun. 2022 Nov 30;13:7392. doi: 10.1038/s41467-022-35198-1 (PMC9712504; doi:10.1038/s41467-022-35198-1)
Supplement: Supplementary file 1 — Supplementary Information [file 41467_2022_35198_MOESM1_ESM.pdf]

Supplementary Information

Cell type diversity in a developing octopus brain

Ruth Styfhals[1,2], Grygoriy Zolotarov[3], Gert Hulselmans[4,5], Katina I. Spanier[4,5], Suresh Poovathingal[5], Ali M. Elagoz[1], Seppe De Winter[4,5], Astrid Deryckere[1†], Nikolaus Rajewsky[3,6], Giovanna Ponte[2], Graziano Fiorito[2], Stein Aerts[4,5], Eve Seuntjens[1*]

1. Laboratory of Developmental Neurobiology, Department of Biology, KU Leuven, Leuven, Belgium
2. Department of Biology and Evolution of Marine Organisms, Stazione Zoologica Anton Dohrn, Naples, Italy
3. Laboratory for Systems Biology of Gene Regulatory Elements, Berlin Institute for Systems Biology, Max Delbrück Center for Molecular Medicine in the Helmholtz Association, Hannoversche Str. 28, 10115 Berlin, Germany
4. Department of Human Genetics, KU Leuven, Leuven 3000, Belgium
5. VIB Center for Brain & Disease Research, KU Leuven, Leuven 3000, Belgium
6. Department of Pediatric Oncology/Hematology, Charité-Universitätsmedizin Berlin, Berlin, Germany

† Current affiliation: Department of Biological Sciences, Columbia University, New York, United States

* Corresponding author: eve.seuntjens@kuleuven.be

This PDF file includes:

Supplementary Figures 1 - 24

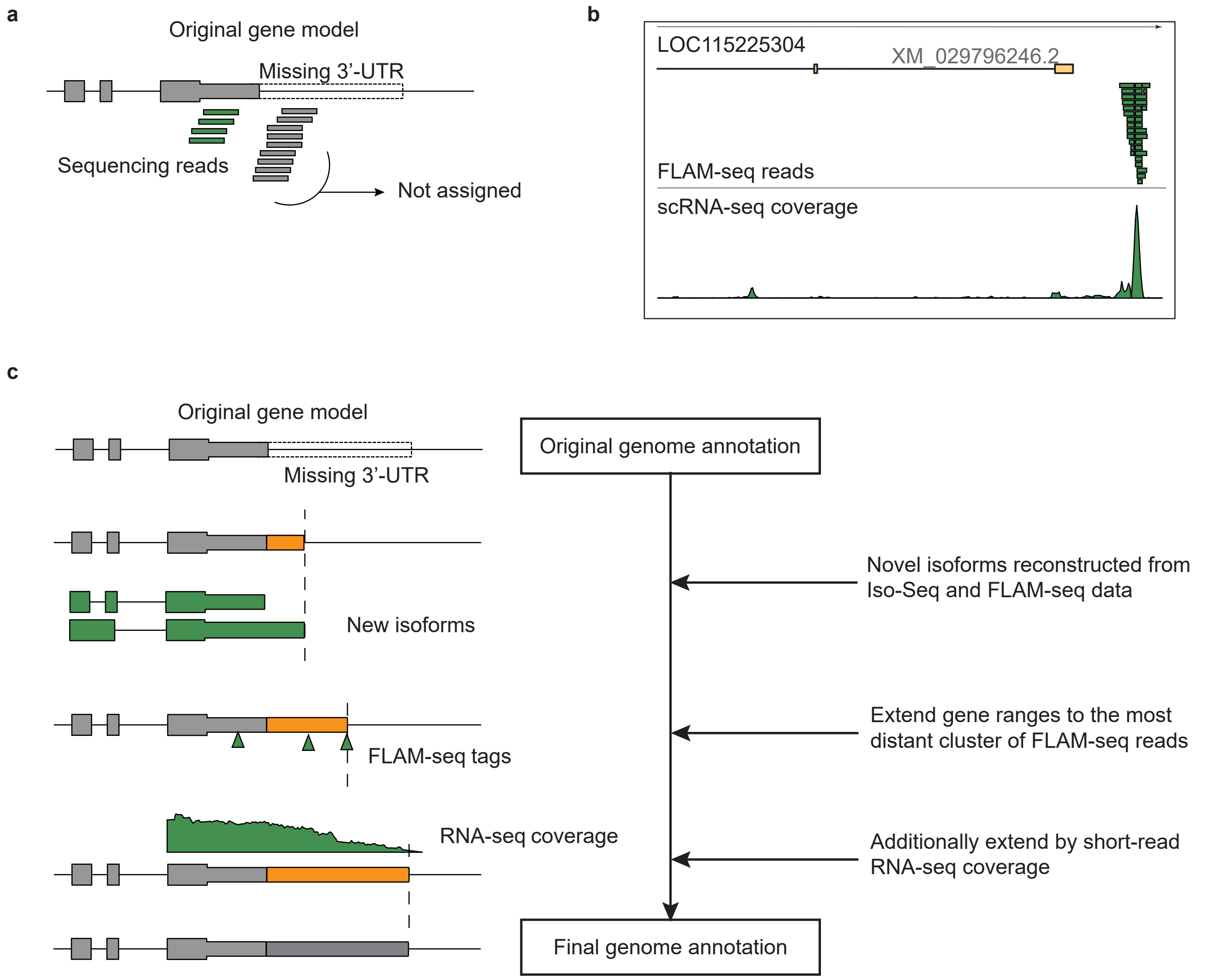

Supplementary Fig. 1 Extending the 3' gene annotation. **a** Hypothetical gene with incomplete 3' UTR annotation. Sequencing reads are not counted. **b** *iqsec2* gene with incomplete 3' UTR annotation. The end of the 3' UTR is shown by the FLAM-seq reads, which coincide with read coverage of sc/snRNA sequencing data. **c** Schematic overview of the methods used to extend the gene annotation.

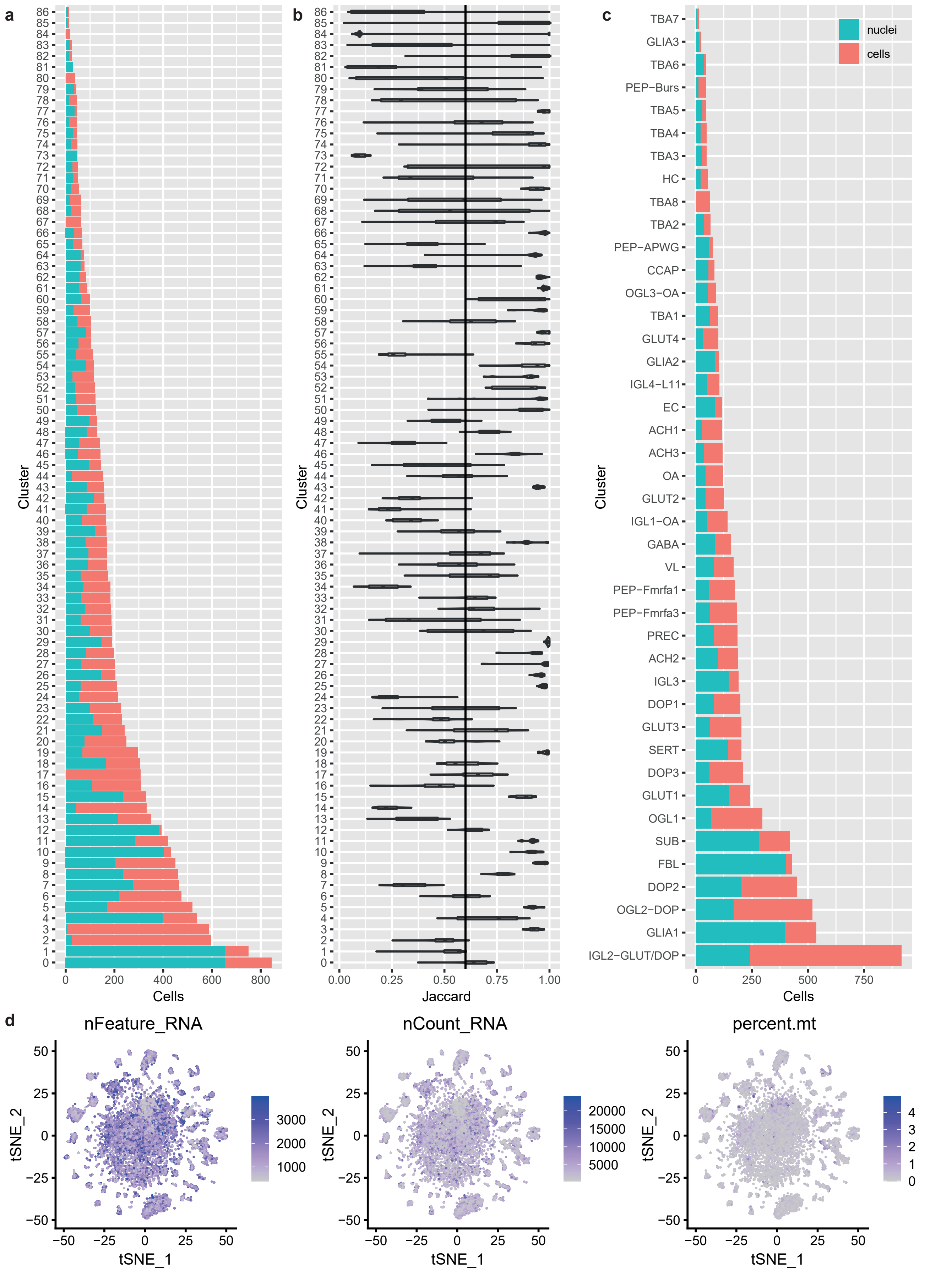

Supplementary Fig. 2 Data integration, clustering and quality control. **a** Resulting clusters after data integration of cells and nuclei. The number of cells originating from either the cells or the nuclei are color coded. **b** Box plot shows median Jaccard indices after subsampling and reclustering with parameters that result in the highest number of stable clusters (dims =150, k.param =10, resolution=2). Box limits represent the first and third quartile medians, whiskers show interquartile intervals, outliers are not shown. Underlying violin plots summarize the distribution of all values (n=20). Clusters with mean Jaccard indices above 0.6 are considered as stable clusters. **c** Resulting stable clusters after data integration between cells and nuclei. The number of cells originating from either the cells or the nuclei are color coded. **d** Multiple metrics for quality control of the resulting dataset are represented on the t-SNE plot.

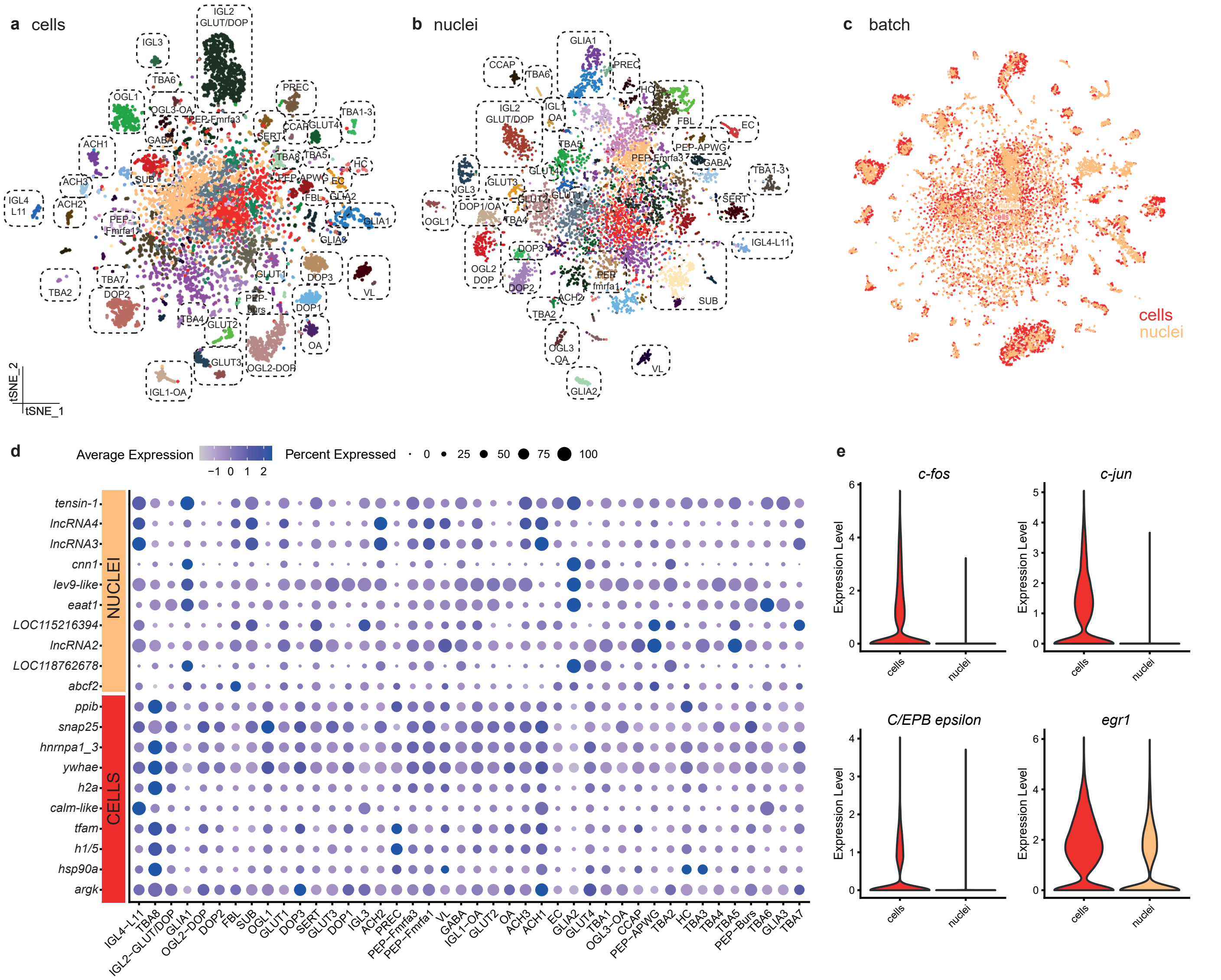

Supplementary Fig. 3 Single cell versus single nuclei RNA sequencing. **a** Cell type annotation of the cells. GLIA2 and FBL were underrepresented. **b** Cell type annotation of the nuclei. GLIA3, ACH3, ACH1 and PEP-burs cell types were mostly absent. **c** Sample identity of all cells in the integrated dataset. **d** Dot plot of top 10 differentially expressed genes between all cells versus nuclei. **e** Violin plots of immediate early genes in cells versus nuclei. ACH, cholinergic neurons; CCAP, cardioactive peptide cells; DOP, dopaminergic neurons; EC, endothelial cells; FBL, fibroblasts; GABA; GABAergic neurons; GLUT, glutamatergic neurons; HC, hemocytes; IGL, inner granular layer cells; OA, octopaminergic neurons; OGL, outer granular layer cells; PEP, peptidergic neurons; PREC; precursors; SERT, serotonergic neurons; SUB, subesophageal neurons; TBA, to be annotated; VL, vertical lobe cells.

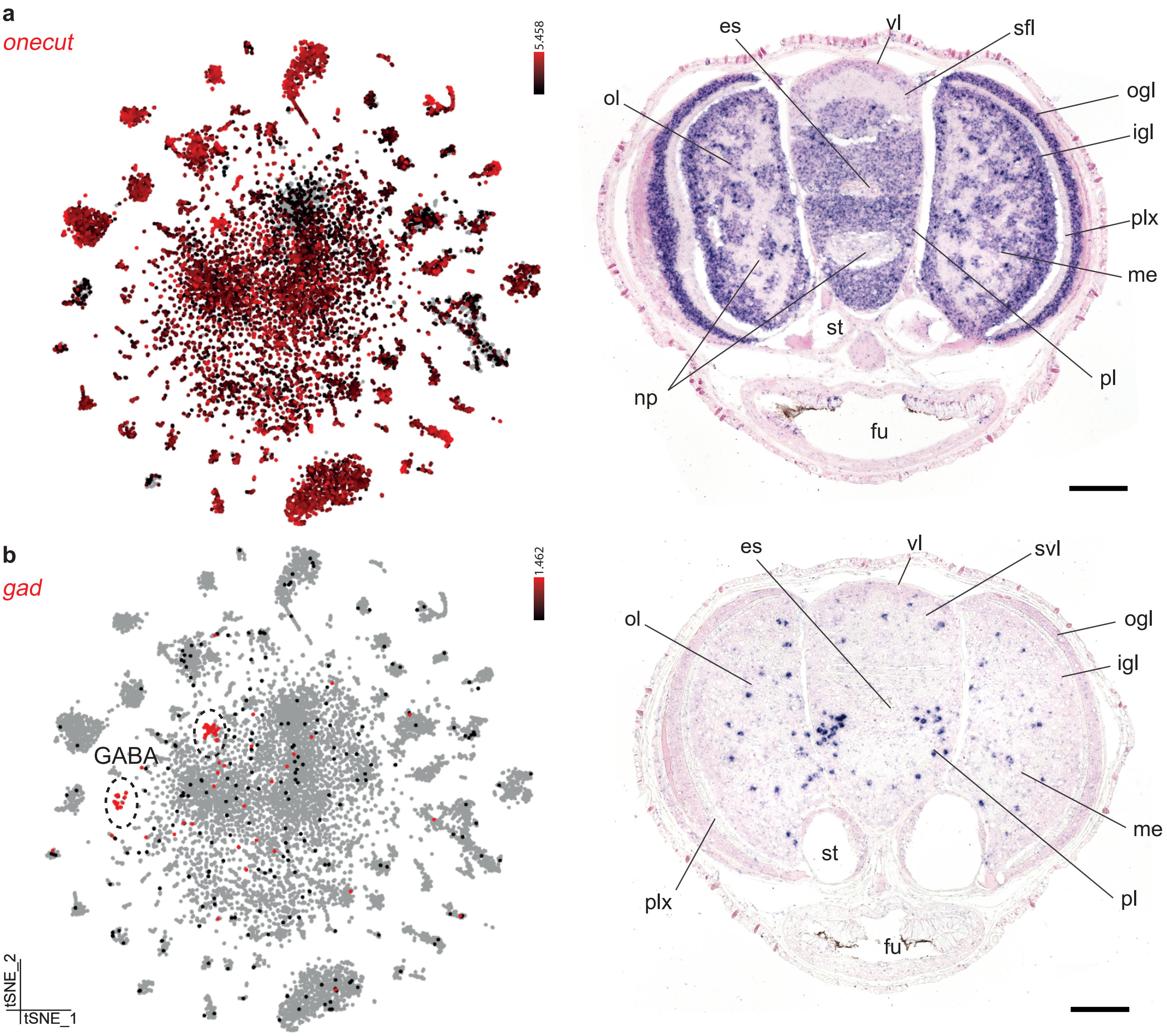

Supplementary Fig. 4 Neuronal markers. **a** *onecut* expression is visualized on a t-SNE plot. *In situ* hybridization for onecut is shown on the right. Perikaryal layers are stained in purple and neuropil (np) is visible in pink. **b** t-SNE representation of GABAergic cells. Expression of Glutamate decarboxylase (*gad*) is shown in red. Scale bars represent 100 µm. es, esophagus; fu, funnel; igl, inner granular layer; me, medulla; np, neuropil; ogl, outer granular layer; ol, optic lobe; pl, pedal lobe; plx, plexiform layer; sfl, superior frontal lobe; st, statocysts; sfl, superior frontal lobe; svl, subvertical lobe; vl, vertical lobe.

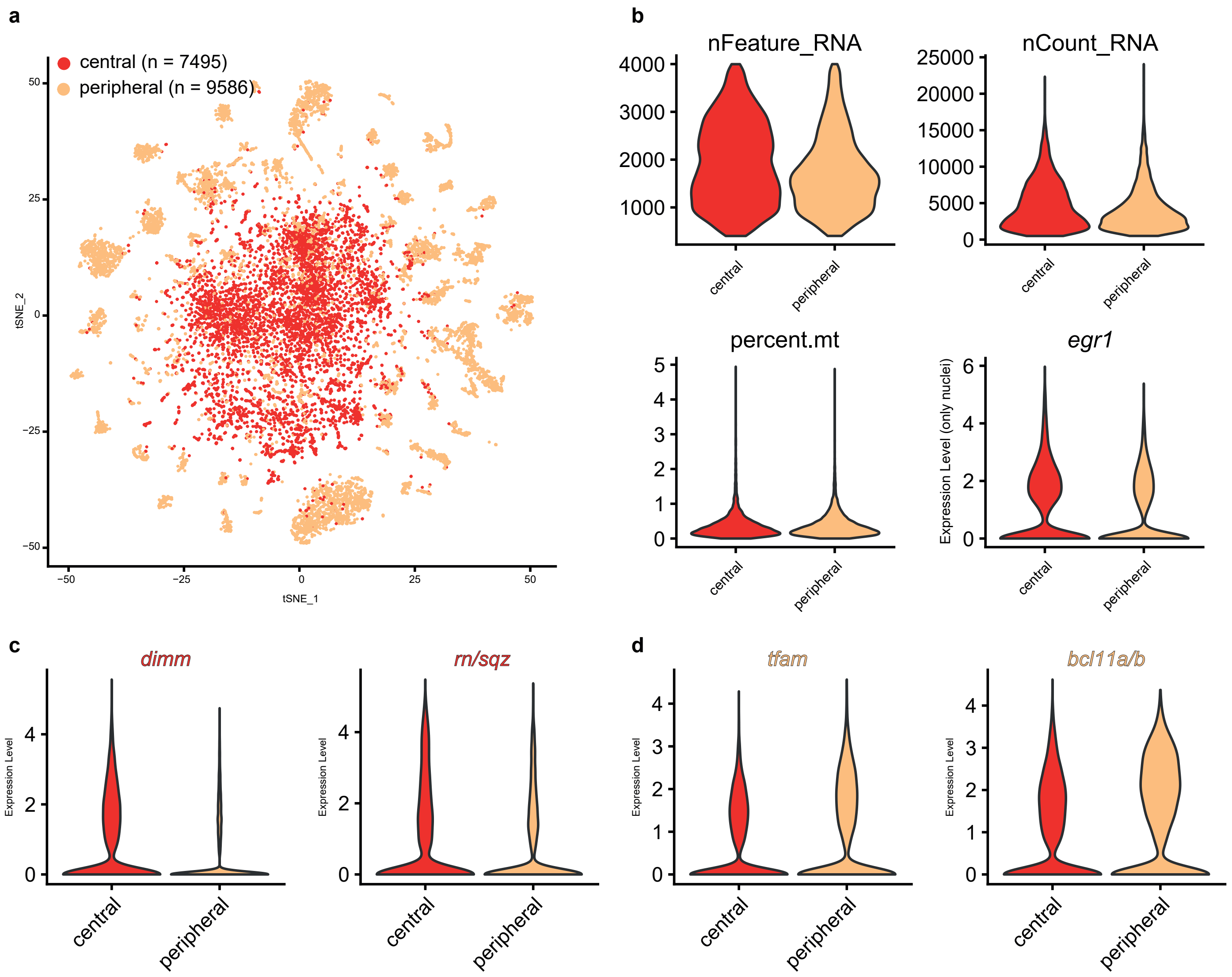

Supplementary Fig. 5 Central versus peripheral cells. **a** Cells were grouped as either central or peripheral depending on their location on the t-SNE plot. **b** Violin plots illustrating quality control metrics for the central versus the peripheral cells. The number of features, counts, percentage of mitochondrial reads and the expression of immediate early gene *egr1* (early growth response 1) are shown. **c** Violin plots of two transcription factors that are highly expressed in the central cells. **d** Violin plot of two transcription factors that are highly expressed in the peripheral cells.

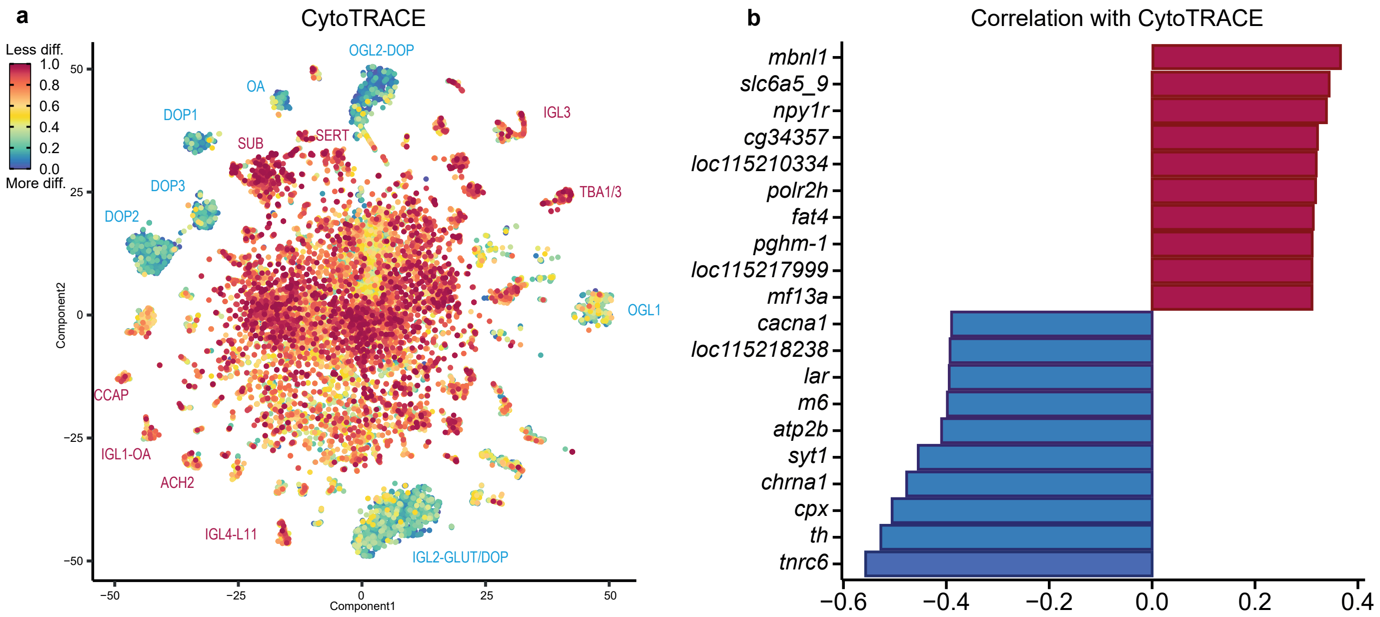

Supplementary Fig. 6 Cellular differentiation based on transcriptional diversity (iCytoTRACE). **a** All neurons were color-coded based on their transcriptional potential and hence differentiation state, and visualized on the t-SNE embedding. **b** Bar plot showing the top 10 genes that were associated with stemness (in red) and differentiation (in blue).

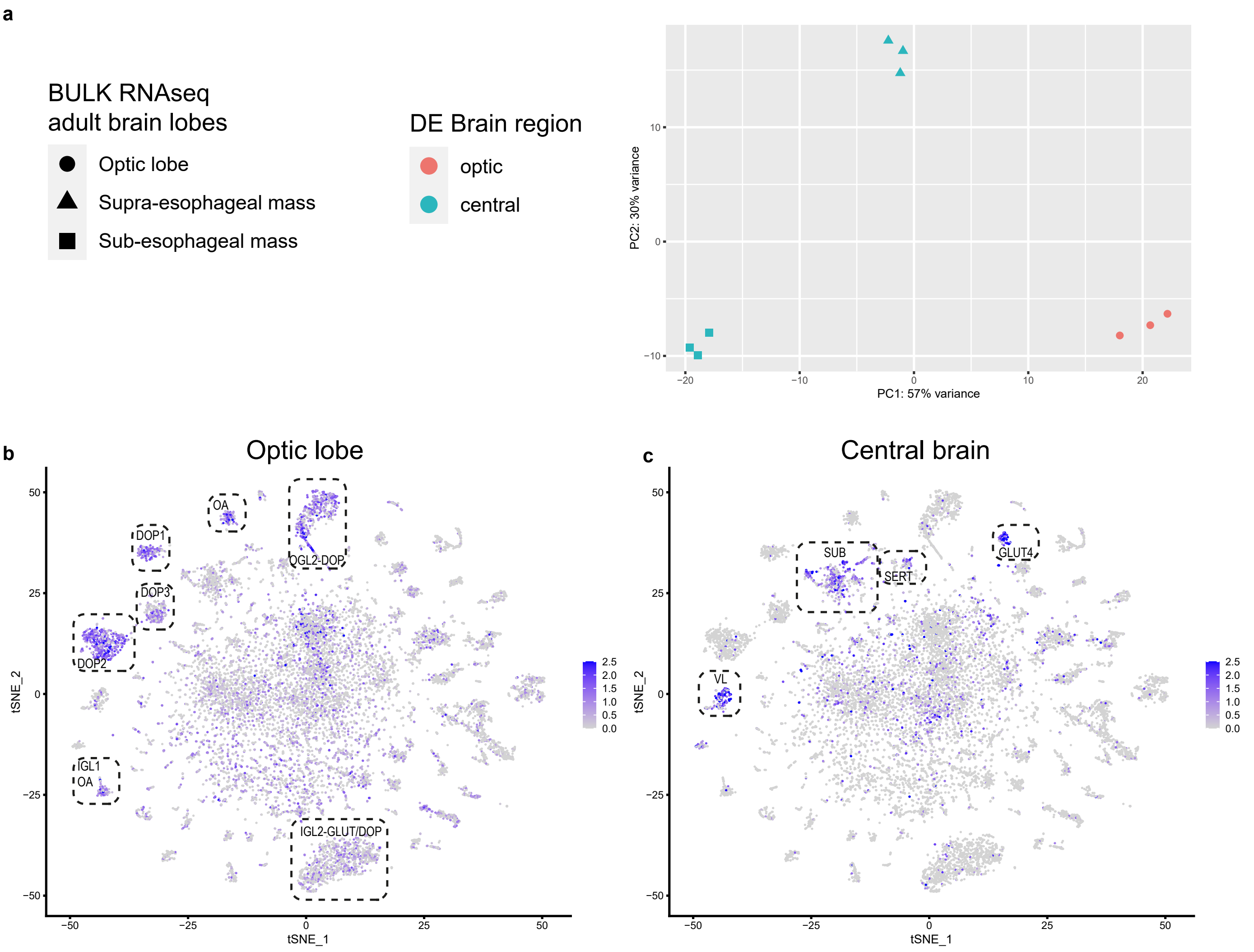

Supplementary Fig. 7 Comparison single cell RNAseq with bulk RNAseq data. **a** Bulk RNA seq data of adult *Octopus vulgaris* brain tissues shown on a PCA plot (samples from Petrosino et al., 2022). **b,c** Top 100 upregulated genes in the optic lobe versus central brain were visualized on the tSNE plot with the AddModuleScore function in Seurat.

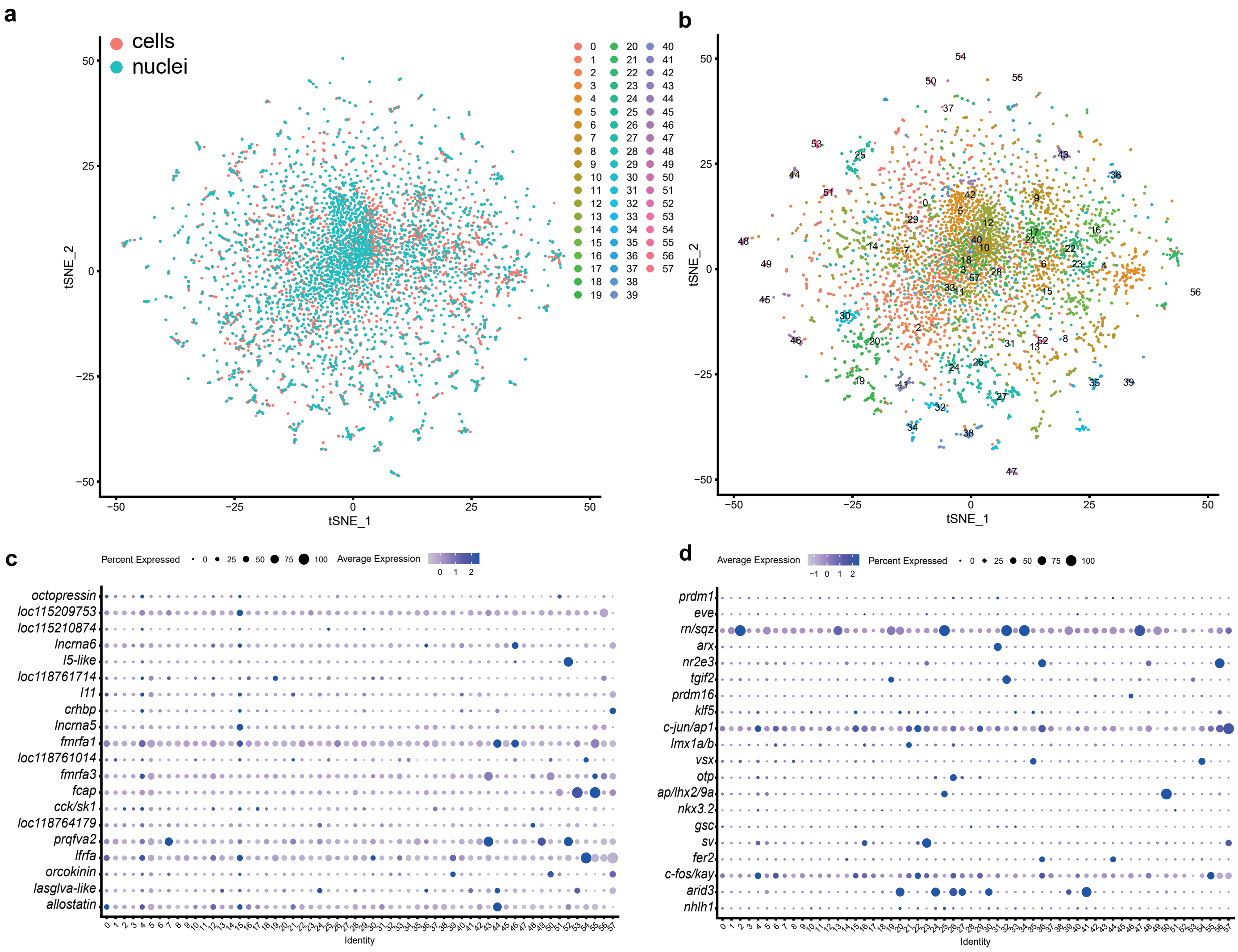

Supplementary Fig. 8 Subclustering of the central constellation. **a** All cells from the central constellation were sub-clustered. **b** 57 clusters were identified. **c** Dot plot illustrating the expression of the top 20 highly variable genes in the central constellation. **d** Dot plot illustrating the expression of the top 20 highly variable transcription factors in the central constellation.

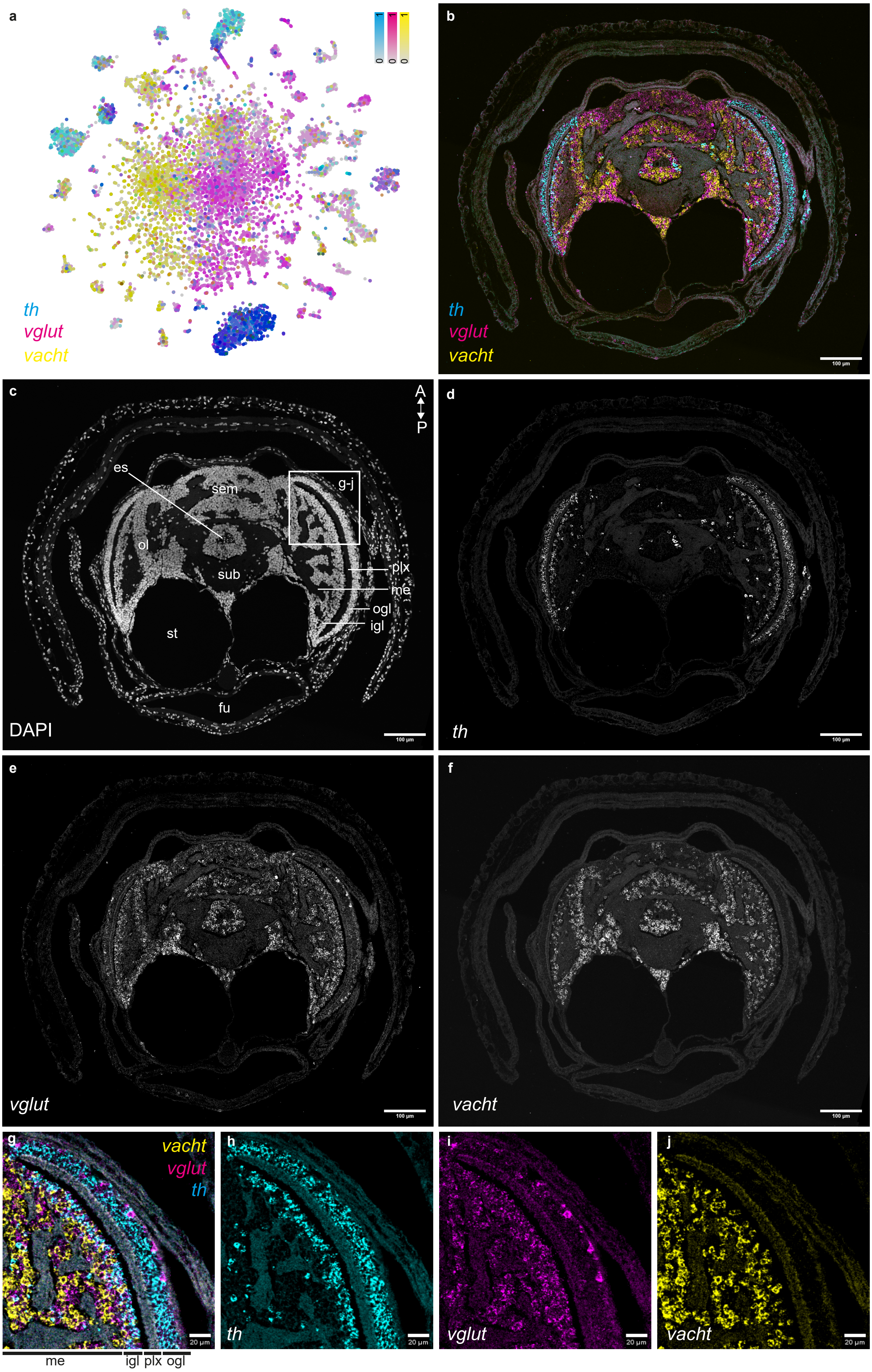

Supplementary Fig. 9 Multiplexed hybridization chain reaction (HCR) of main neurotransmitters. **a** t-SNE representation of th, vglut, and vacht co-expression in CMY colors. **b** HCR merge in CMY colors without DAPI. **c** DAPI with anatomical annotations. The white square indicates the magnification shown in g-j. In **d, e, f** separate channels are shown in grey scale. **g** Magnification HCR merge without DAPI. **h-j** separate channels are shown. A, anterior; es, esophagus; fu, funnel; igl, inner granular layer; me, medulla; ogl, outer granular layer; ol, optic lobe; plx, plexiform layer; P, Posterior; sub, subesophageal mass; sem, supra-esophageal mass; st, statocysts.

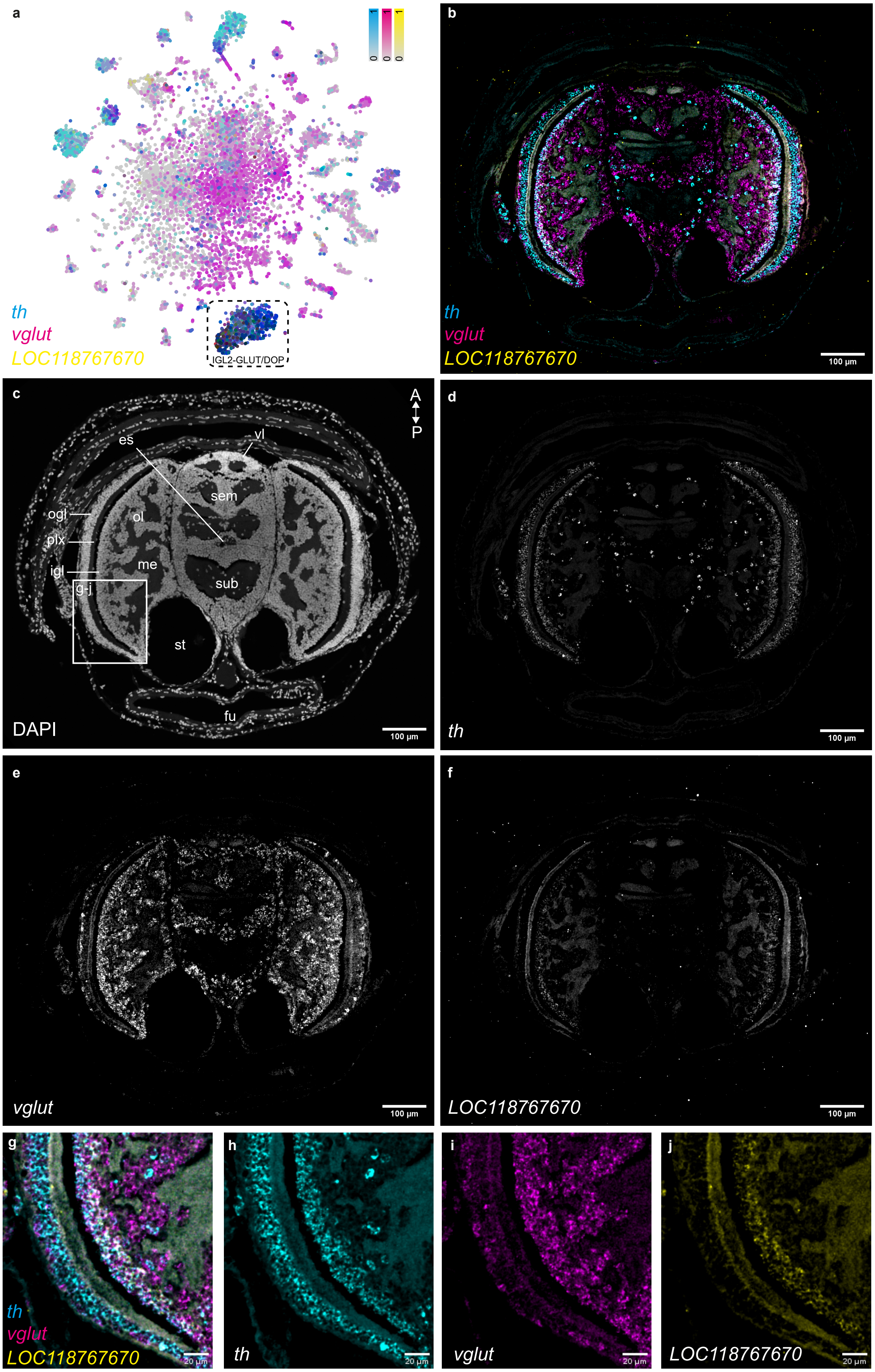

Supplementary Fig. 10 Multiplexed hybridization chain reaction (HCR) visualizing IGL2-GLUT/DOP neurons. **a** t-SNE representation of *th*, *vglut*, and *LOC118767670* co-expression in CMY colors. **b** HCR merge in CMY colors without DAPI. **c** DAPI with anatomical annotations. The white square indicates the magnification shown in g-j. In **d, e, f** separate channels are shown in grey scale. **g** Magnification HCR merge without DAPI. Overlap can be observed as white in the igl. **h-j** separate channels are shown. A, anterior; es, esophagus; fu, funnel; igl, inner granular layer; me, medulla; ogl, outer granular layer; ol, optic lobe; plx, plexiform layer; P, Posterior; sub, subesophageal mass; sem, supra-esophageal mass; st, statocysts; vl, vertical lobe.

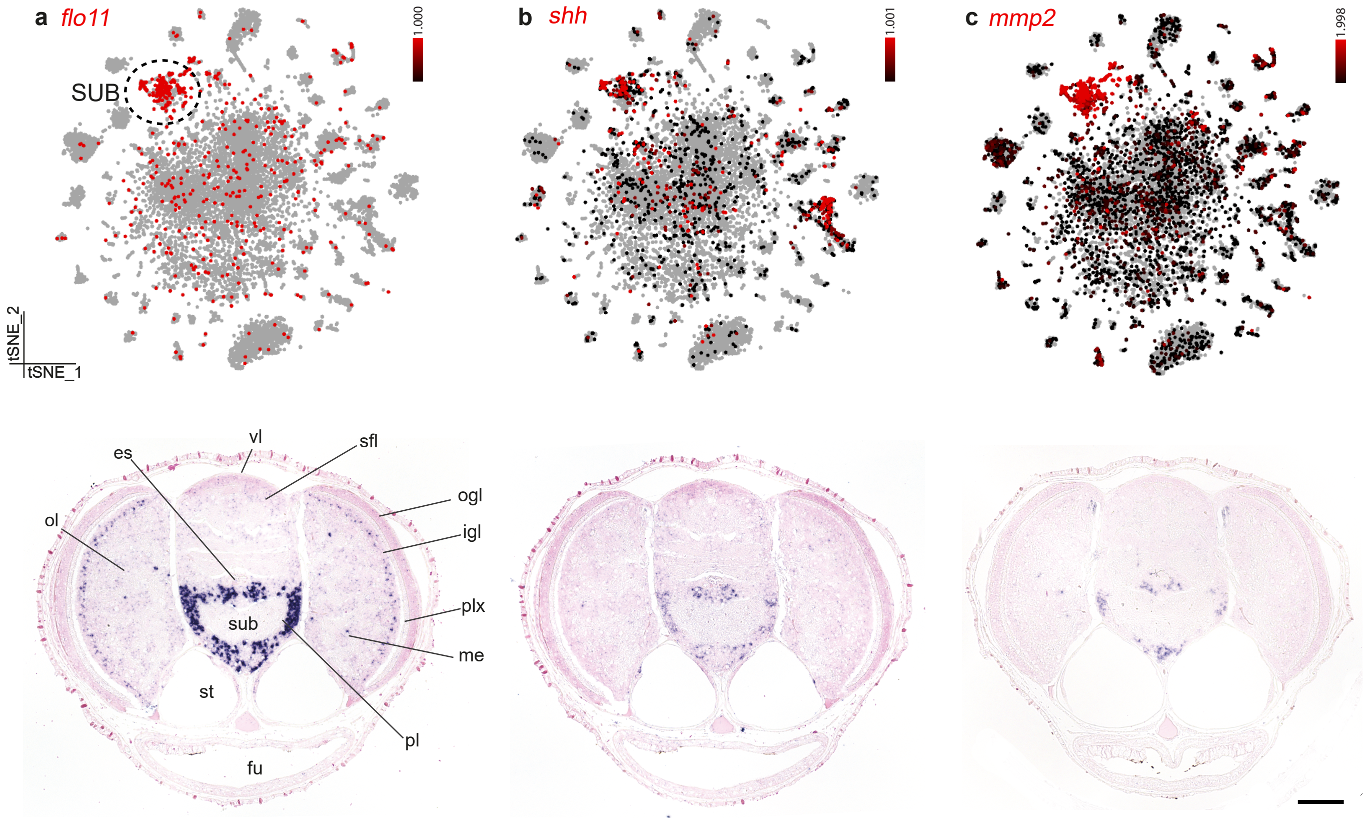

Supplementary Fig. 11 Sub-esophageal lobe cells. Expression of *flo11*, *shh* and *mmp2* is visualized in a t-SNE plot in a, b, c. **a** Flocculation protein-like (*flo11*) is highly expressed within the pedal lobe (pl) of the sub-esophageal mass and in a smaller population in the igl. **b** Sonic hedgehog (*shh*) is expressed in the sub-esophageal mass and in subpopulation of the glial cells. **c** Matrix metallopeptidase 2 (*mmp2*) is expressed in the sub-esophageal mass. igl, inner granular layer; SUB, sub-esophageal mass. Scale bar represents 100 µm. es, esophagus; fu, funnel; igl, inner granular layer; me, medulla; ogl, outer granular layer; ol, optic lobe; pl, pedal lobe; plx, plexiform layer; sfl, superior frontal lobe; st, statocysts; vl, vertical lobe.

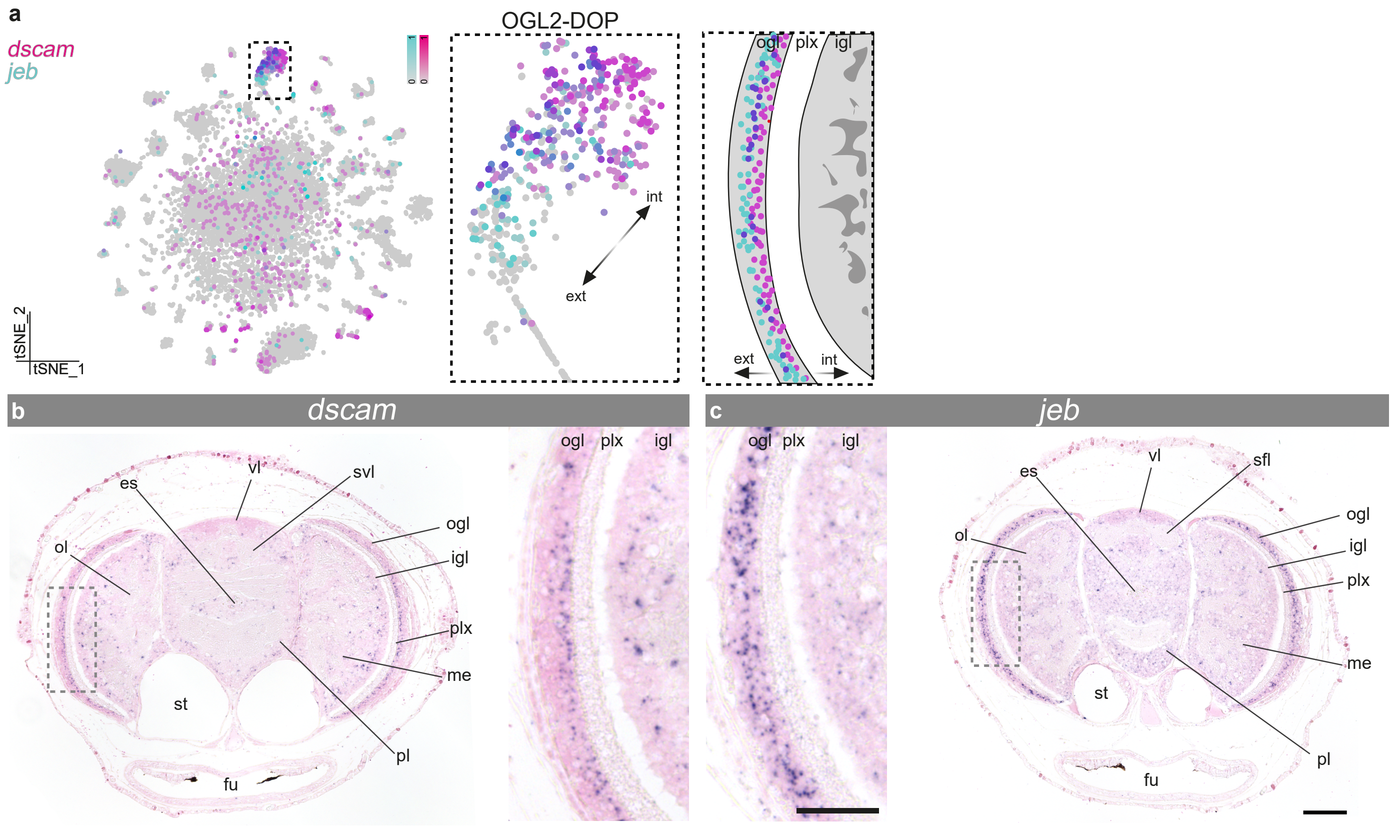

Supplementary Fig. 12 Dopaminergic cells in the outer granular layer. **a** t-SNE representation of two different markers for the OGL2-DOP population. *dscam* (Down syndrome cell adhesion molecule) is shown in magenta and *jeb* (Jelly belly) in cyan. A magnification for OGL2-DOP is shown together with a color coded scheme for *jeb* and *dscam* expression. **b** *dscam* is expressed in small cells more towards the interior of the outer granular layer. **c** Neuropeptide *jeb* is expressed throughout the ogl in dopaminergic neurons. Magnified regions are annotated with a grey box. Scale bars for the overview images represent 100 µm and for the magnifications, 50 µm. es, esophagus; fu, funnel; igl, inner granular layer; me, medulla; ogl, outer granular layer; ol, optic lobe; pl, pedal lobe; plx, plexiform layer; sfl, superior frontal lobe; svl, subvertical lobe; st, statocysts; vl, vertical lobe.

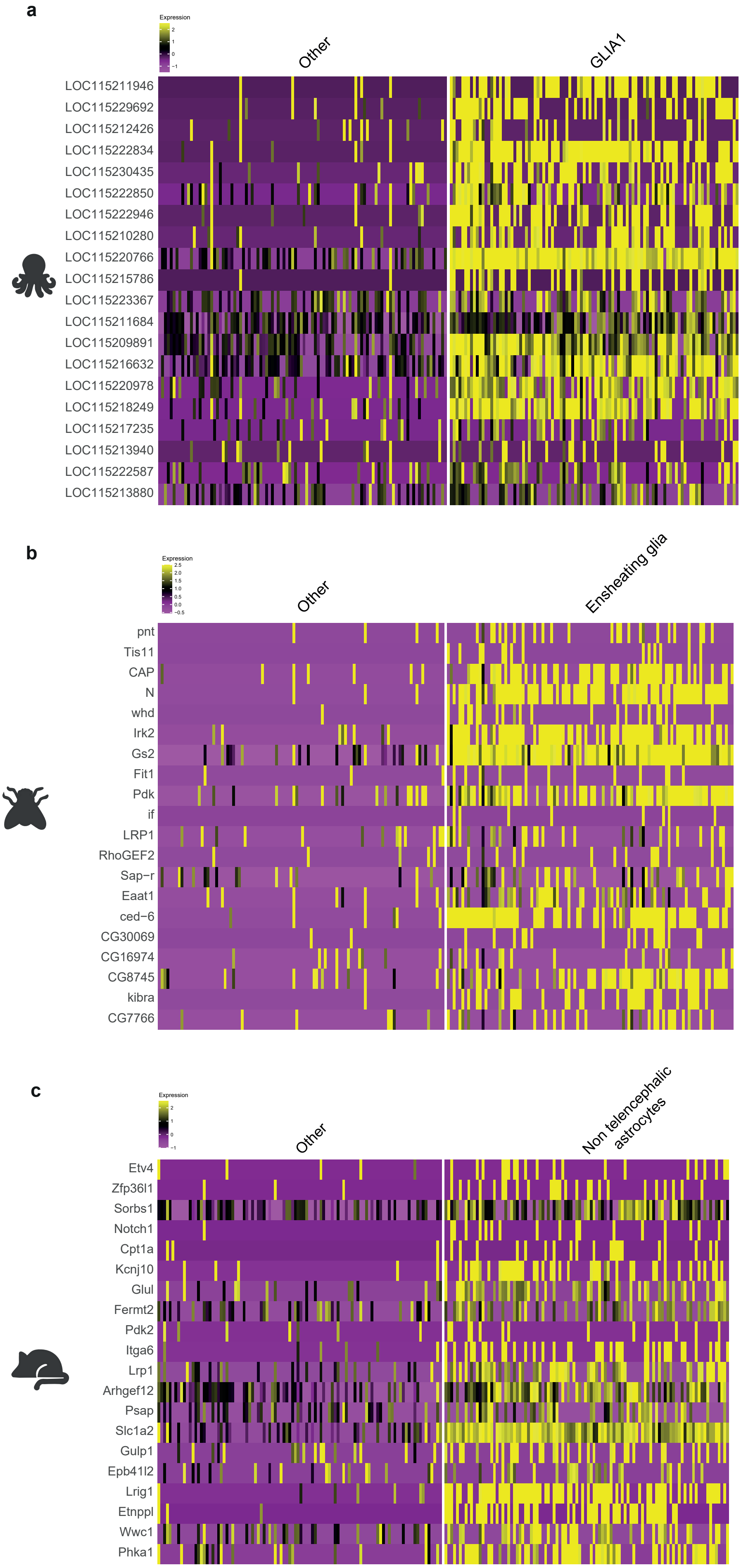

Supplementary Fig. 13 Conserved glial gene expression signature across species based on putative one-to-one orthologues. Octopus genes are shown in **a** (this study), fly in **b** (Davie *et al.*, 2018), and mouse in **c** (Kleshchevnikov *et al.*, 2020). Expression of all other cell types identified in the study is grouped under “Other”.

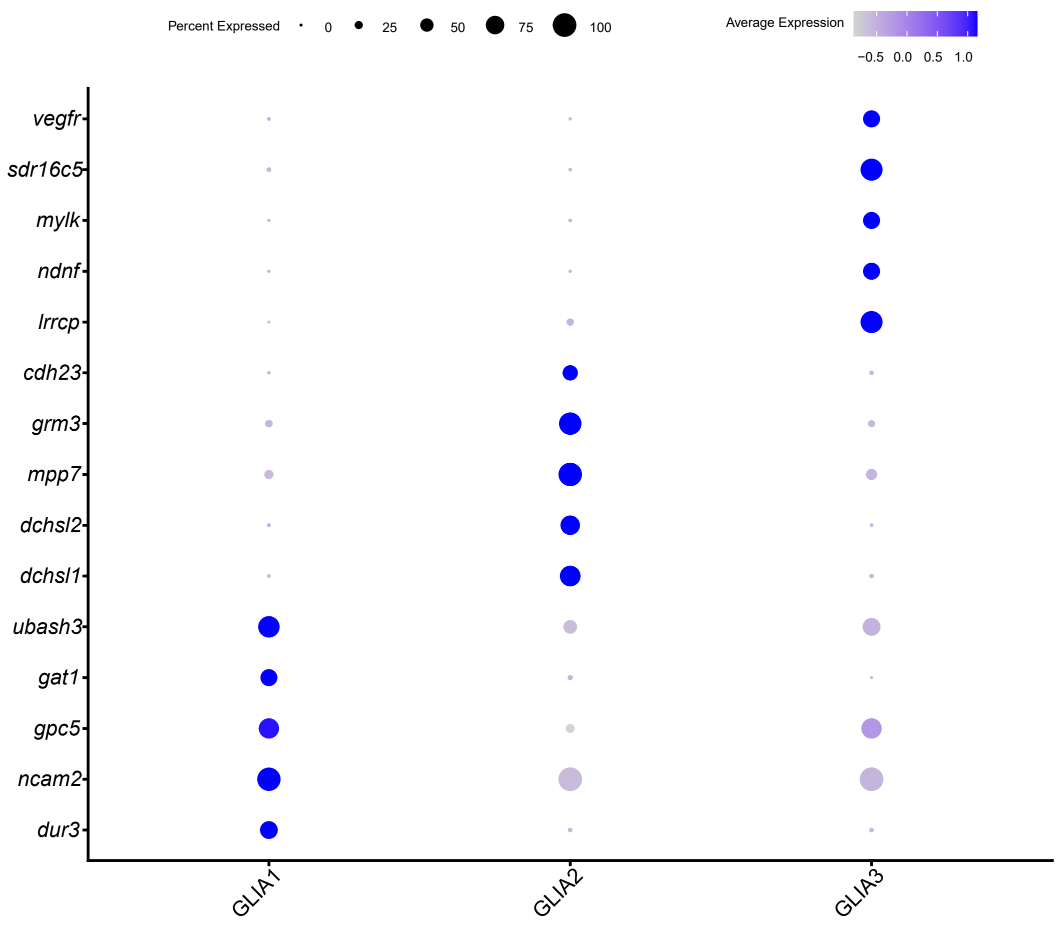

Supplementary Fig. 14 Glial subtypes. Dot plot visualizing the expression of the top 5 differentially expressed genes between glial subtypes; GLIA1, 2, 3.

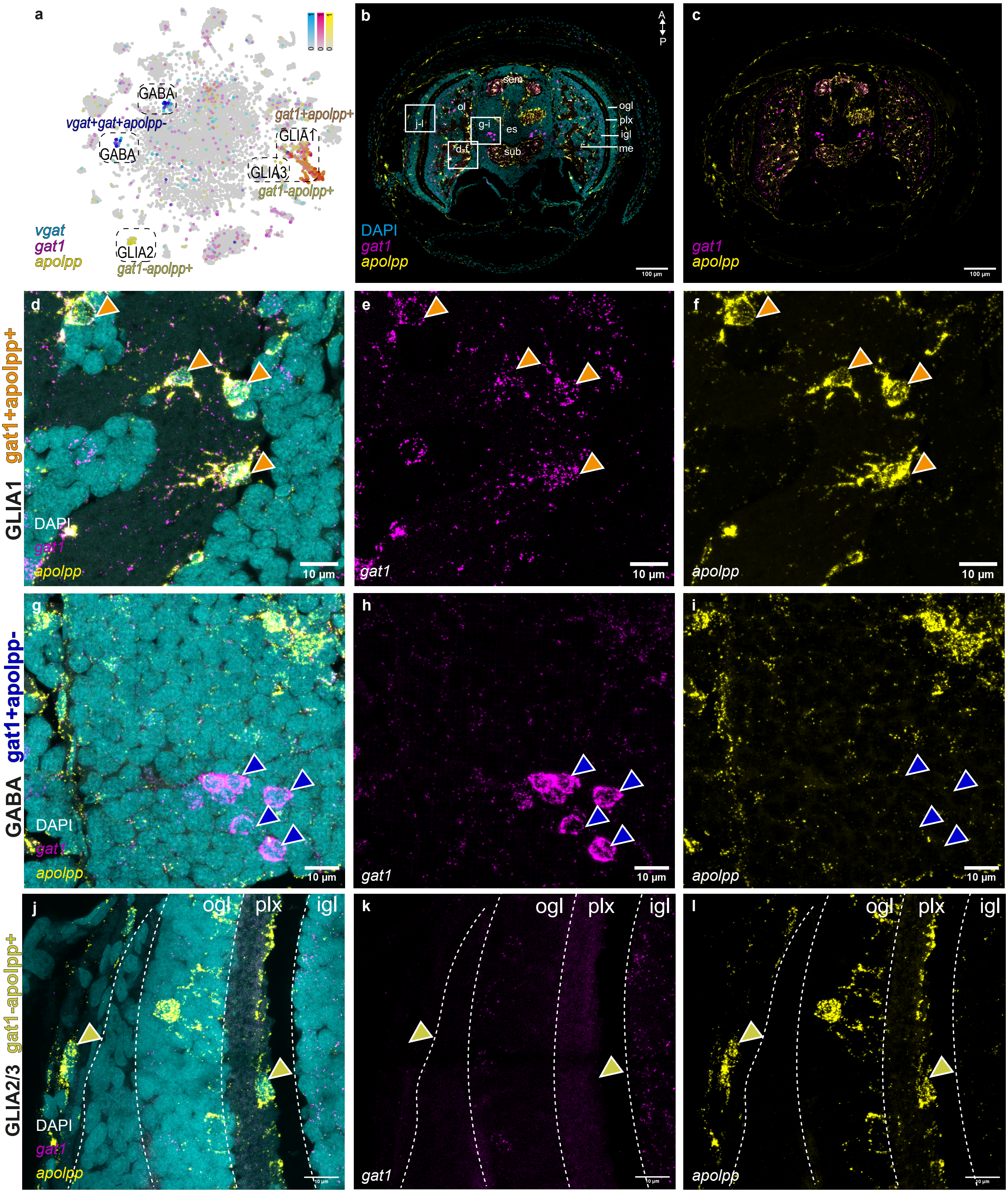

Supplementary Fig. 15 Glial subtypes in the octopus brain. **a** t-SNE representation of expression levels shown in CMY colors of vesicular GABA transporter (*vgat*, marker for GABAergic neurons), *gat1* (GABAergic transporter 1, expressed in GABAergic neurons and GLIA1), *apolpp* (apolipoprotein that is expressed in all glial populations). **b** Overview image of a transversal brain section of a one day old paralarva. Multiplexed hybridization chain reaction for *apolpp* (shown in yellow) and *gat1* (shown in magenta) combined with a nuclear stain DAPI (shown in cyan). White squares indicate higher magnifications shown in d-f, g-i and j-l. **c** same as b without dapi to visualize coexpression. **d-f** 60x magnification of cells belonging to the GLIA1 subtype that co-express *gat1* and *apolpp* (indicated by orange arrows). **g-i** 60x magnification of GABAergic neurons indicated by dark blue arrows (express *gat1* but not *apolpp*). **j-l** 60x magnification of glial cells belonging to GLIA2/3 that express *apolpp* but not *gat1* (yellow arrows). es, esophagus; igl, inner granular layer; me, medulla; ogl, outer granular layer; ol, optic lobe; plx, plexiform layer; sem, supra-esophageal mass; sub, sub-esophageal mass.

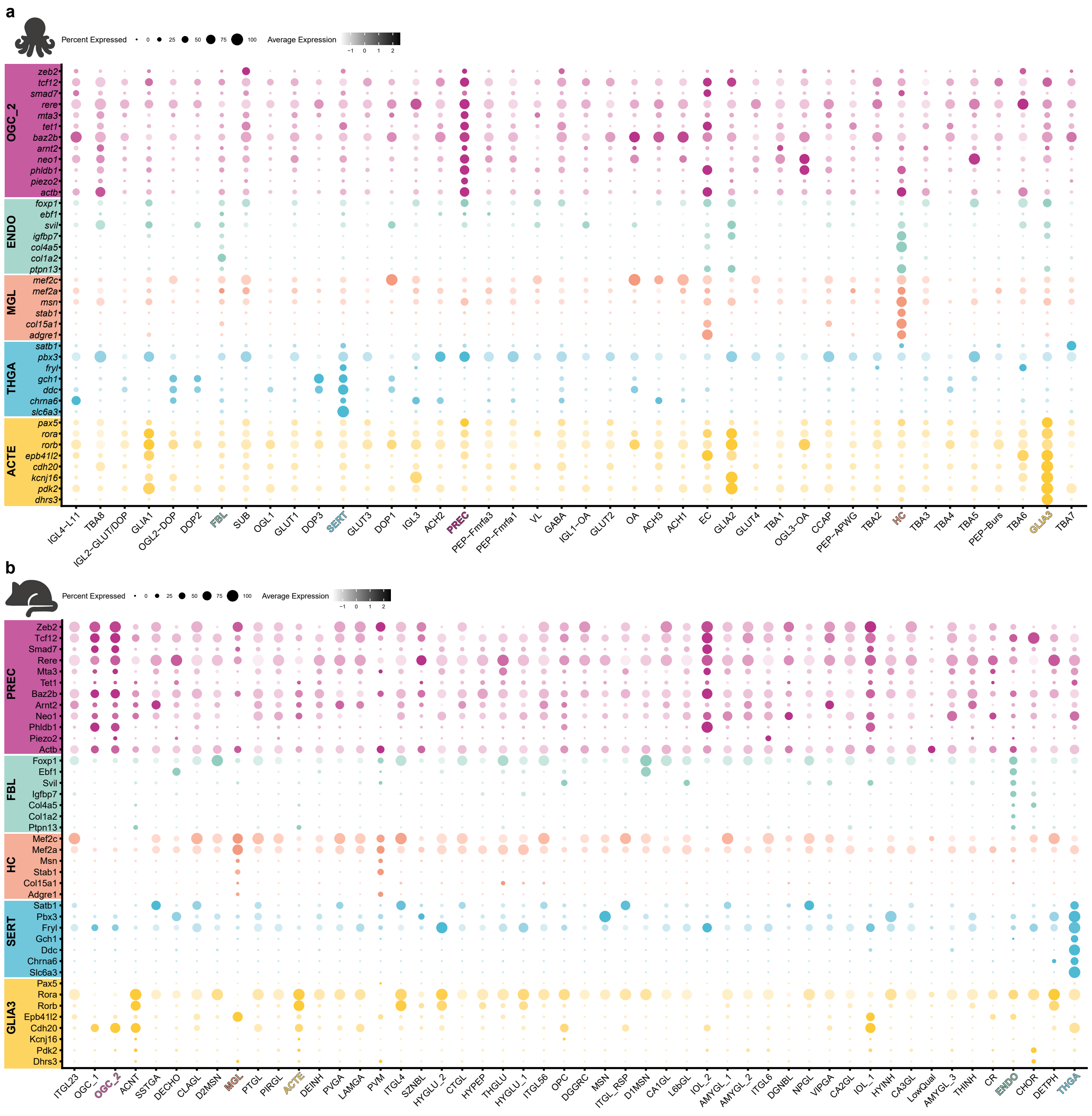

Supplementary Fig. 16 Marker genes and transcription factors underlying cell type mappings between octopus and mouse. **a** Dot plot of expression levels of octopus genes. **b** Dot plot of expression levels of *M. musculus* genes (data from Kleshchevnikov , 2020). Gene lists responsible for each cell type mapping were sorted based on high expression in octopus and the top 5 best reciprocal BLAST hits were plotted. These gene lists were also filtered for transcription factors, which are also visualized in the dot plots above. ACTE, telencephalic astrocytes; ENDO, endothelial cells; FBL, fibroblasts; GLIA3, glia 3 subtype; HC, hemocytes; MGL, microglia; OGC_2, oligodendrocytes; PREC, precursors; SERT, serotonergic neurons; THGA, dopaminergic interneurons. No data is shown for GLIA1 and non-telencephalic astrocytes (ACNT) since this is shown in Supplementary Fig. 13. Color code and cell type annotation in the left sidebar indicate the putative homologous cell types in mouse (a) and octopus (b).

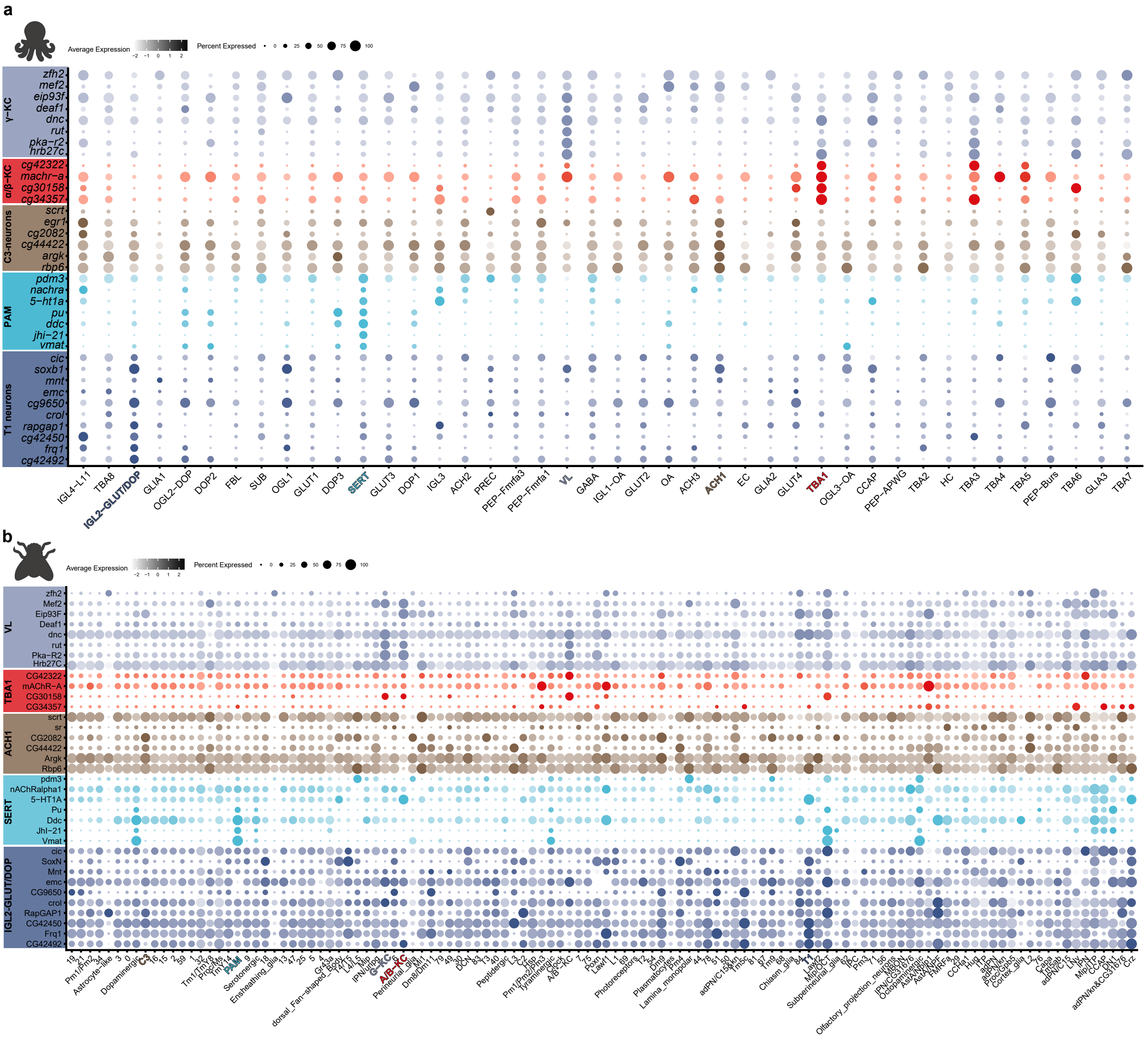

Supplementary Fig. 17 Marker genes and transcription factors underlying cell type mappings between octopus and fly. **a** Dot plot of expression levels of octopus genes. **b** Dot plot of expression levels of *D. melanogaster* genes (data m Davie , 2018). Gene lists responsible for each cell type mapping were sorted based on high expression in octopus and the top 5 best reciprocal BLAST hits were plotted. These gene lists were also filtered for transcription factors, which are also visualized in the dot plots above. α/β-KC, alpha/beta-kenyon cells; γ-KC, gamma-kenyon cells; IGL2-GLUT/DOP, glutamatergic-dopaminergic neurons in the inner granular layer; PAM, protocerebral anterior medial cluster dopamine neurons; SERT, serotonergic neurons; TBA1, to be annotated1; VL, vertical lobe. No data is shown for GLIA1 and ENS (ensheathing glia) since this is shown in Supplementary Fig. 13. Color code and cell type annotation in the left sidebar indicate the putative homologous cell types in fly (a) and octopus (b).

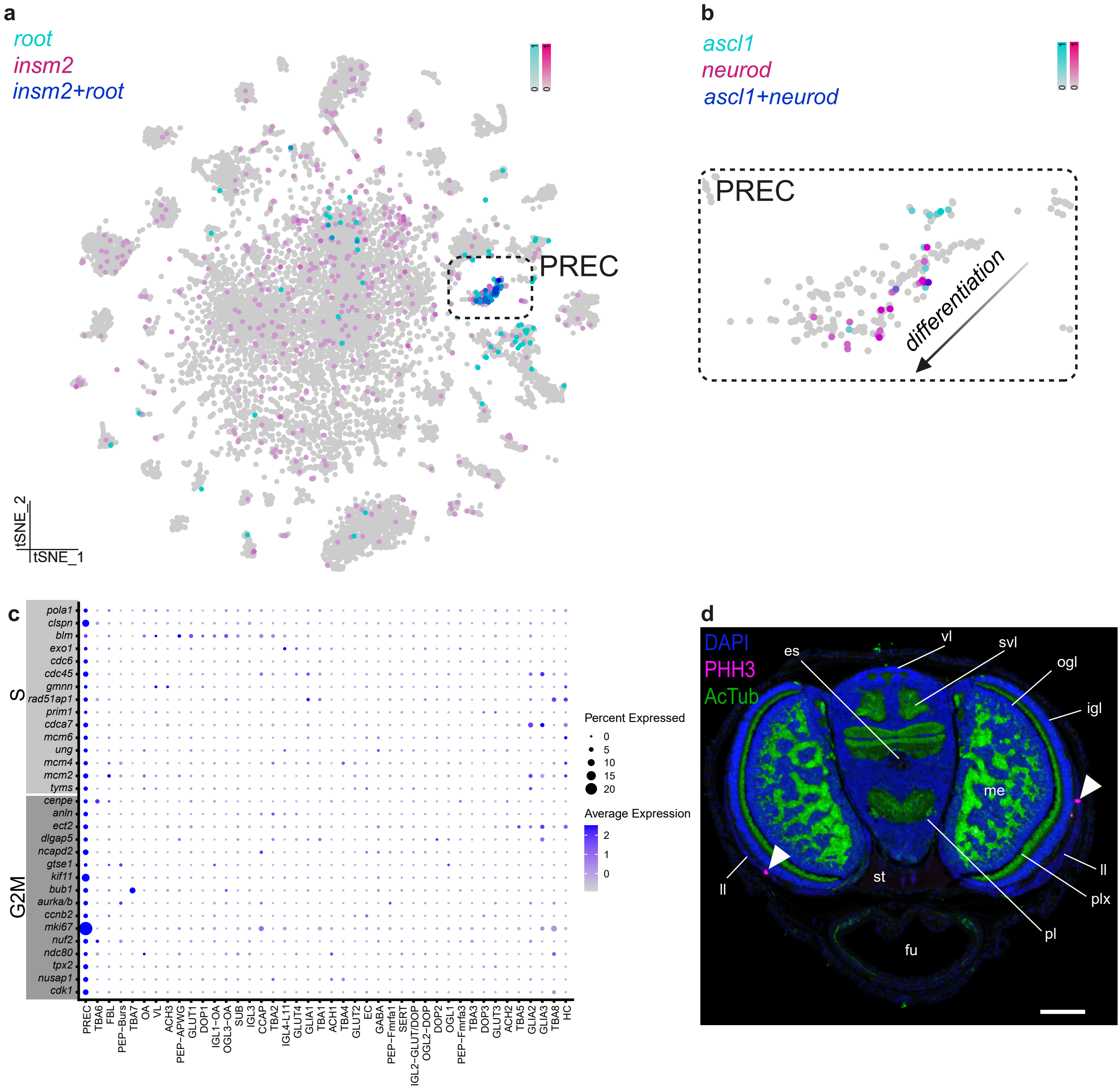

Supplementary Fig. 18 Precursor cells and cell cycle activity in one-day-old paralarva. **a** Expression of Rootletin (*root*) is shown in cyan and Insulinoma-Associated 2 transcriptional repressor (*insm2*) in magenta on the t-SNE plot. **b** Precursors express *ascl1* and *neurod*. A gradient in differentiation is visible. **c** Known markers for G2/M and S phases of the cell cycle are visualized on a dot plot. **d** Phospho-histone H3 (PHH3) staining on a one-day-old paralarva, together with axonal marker acetylated tubulin (AcTub). Cells undergoing mitosis (late G2 and M-phase) are annotated with a white arrow. Scale bar represents 100 μm. es, esophagus; fu, funnel; igl, inner granular layer; ll, lateral lips; ogl, outer granular layer; pl, pedal lobe; plx, plexiform layer; st, statocysts; svl, subvertical lobe; vl, vertical lobe.

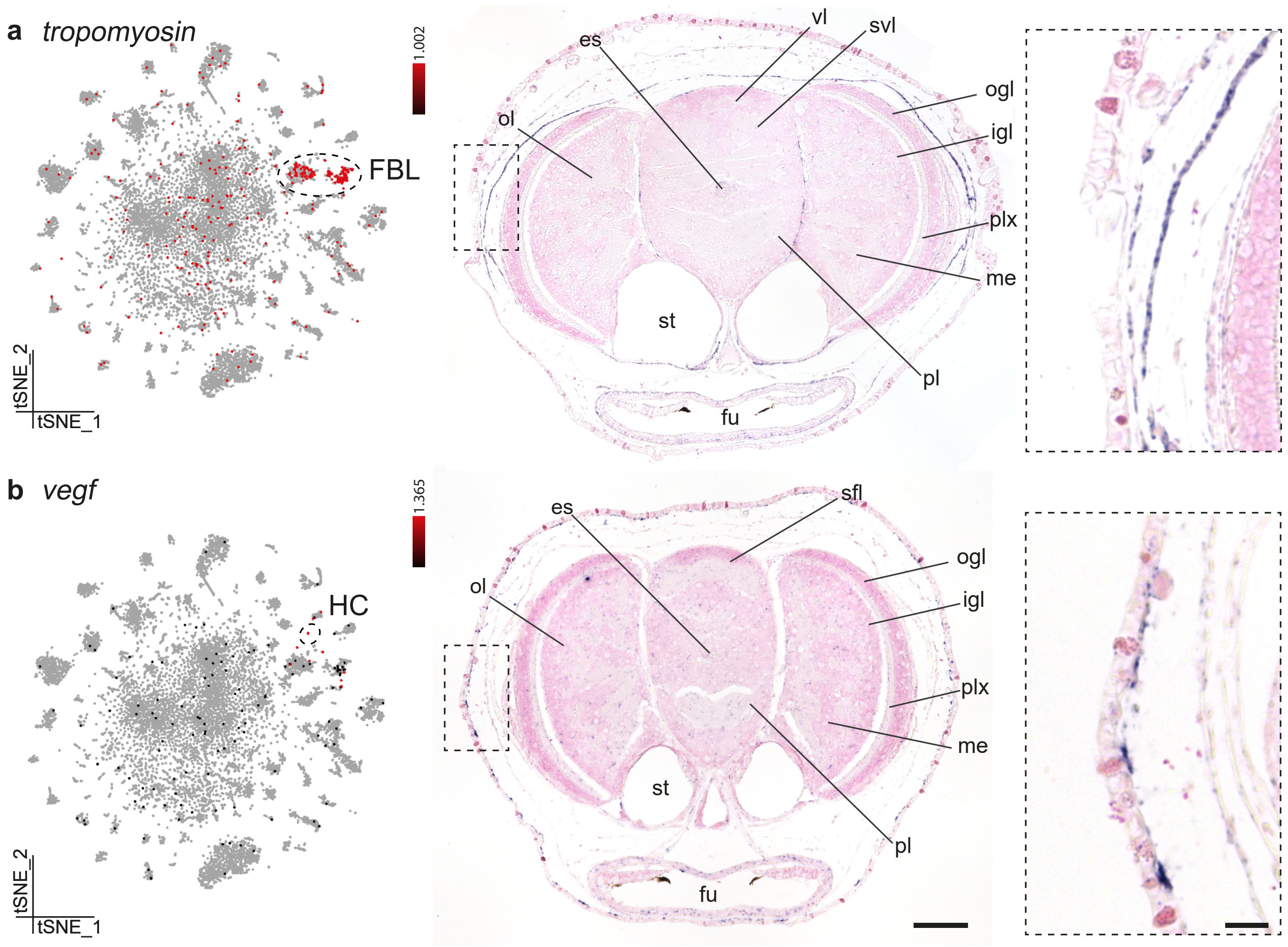

Supplementary Fig. 19 Non-neuronal cell types. **a** *tropomyosin* is expressed within the fibroblasts (FBL). These cells form a layer that surrounds the brain. Expression can also be observed between the optic lobes and the central brain, and surrounding the statocysts. **b** *vegf* (vascular endothelial growth factor) is expressed within the hemocytes (HC). *vegf* expression is visible in a punctuate pattern underneath the epidermis. Scale bars for the overview images represent 100 µm and for the magnifications, 50 µm. es, esophagus; fu, funnel; igl, inner granular layer; me, medulla; ogl, outer granular layer; ol, optic lobe; pl, pedal lobe; plx, plexiform layer; svl, subvertical lobe; sfl, superior frontal lobe; st, statocysts; vl, vertical lobe.

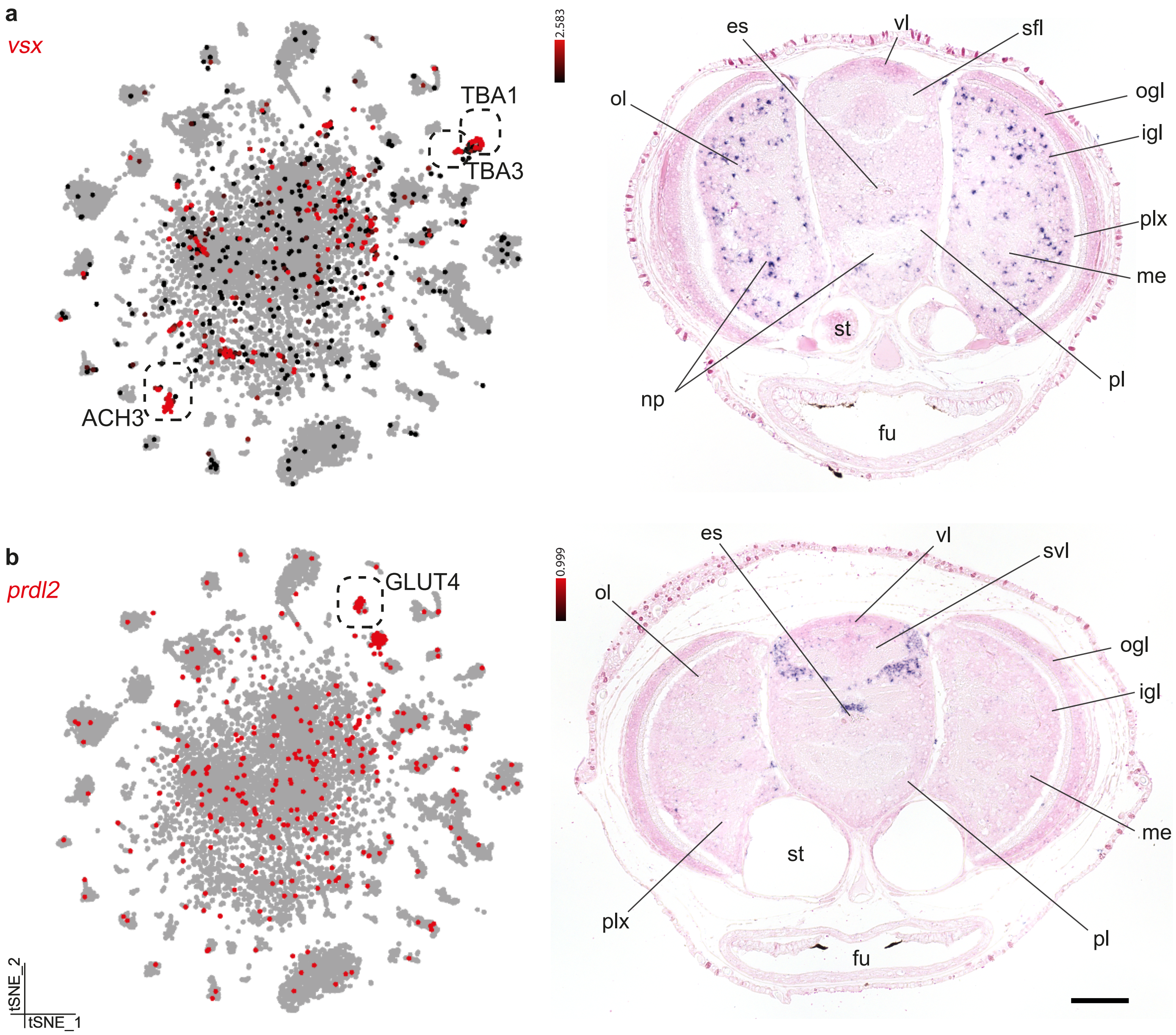

Supplementary Fig. 20 Gene expression profile of Homeobox transcription factors. **a** Visual system Homeobox transcription factor (*vsx*) expression is shown on the tSNE plot together with its *in situ* hybridization. *vsx* is expressed in TBA1, TBA3 and ACH3. These cells are distributed along the medulla of the optic lobes. Some expression is also visible within the sub-esophageal mass. **b** Paired homeobox protein-2-like (*prdl2*) expression is limited to the GLUT4 cluster and an unstable cluster (as shown on the tSNE on the left). With hybridization we map these glutamatergic neurons to clusters of cells within the sub-vertical lobe. Scale bars represent 100 μm. es, esophagus; fu, funnel; igl, inner granular layer; me, medulla; ogl, outer granular layer; ol, optic lobe; pl, pedal lobe; plx, plexiform layer; svl, subvertical lobe; sfl, superior frontal lobe; st, statocysts; svl, subvertical lobe; vl, vertical lobe.

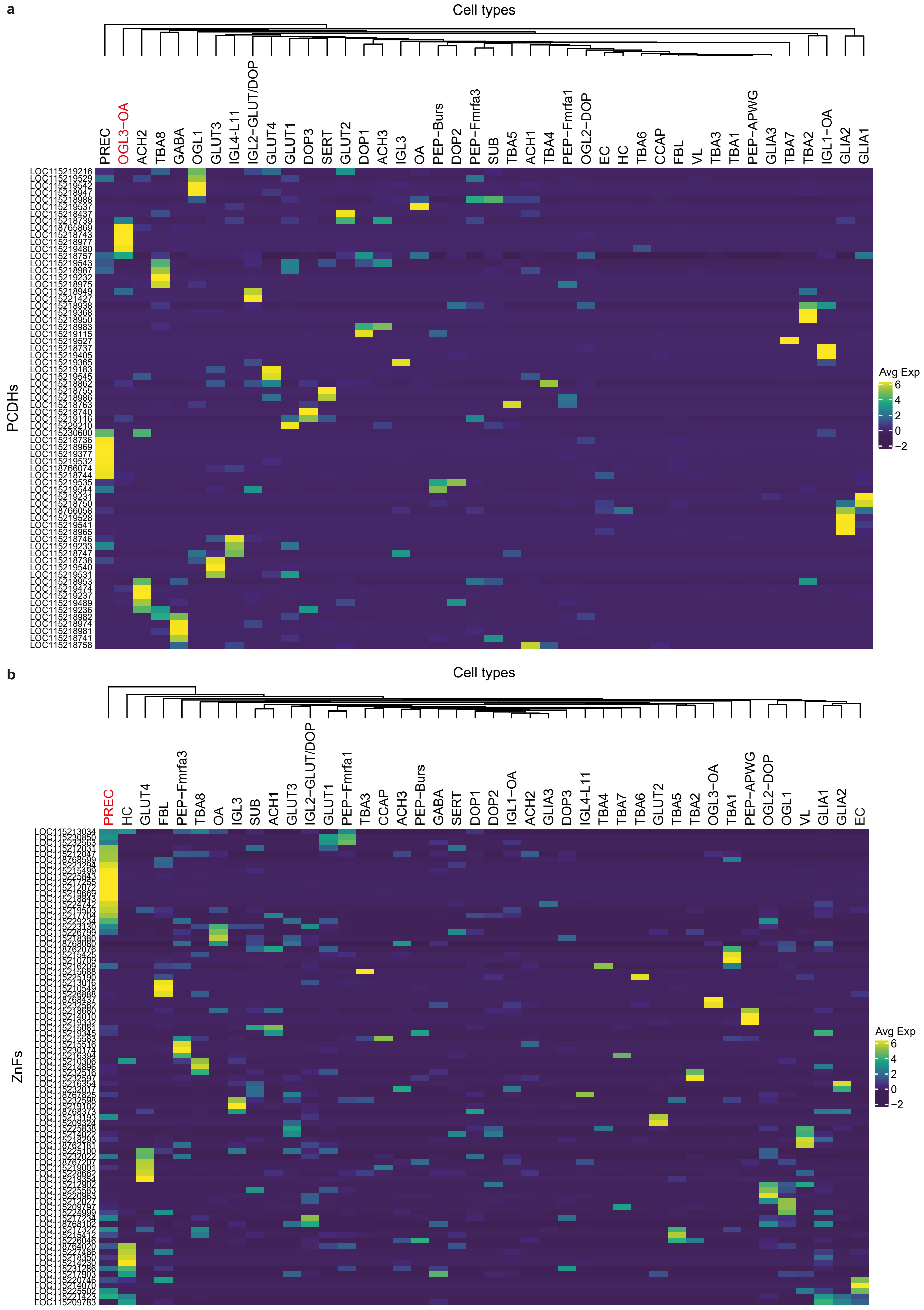
a
Cell types
PREC
OGL3−OA
ACH2
TBA8
GABA
OGL1
GLUT3
IGL4−L11
IGL2−GLUT/DOP
GLUT4
GLUT1
DOP3
SERT
GLUT2
DOP1
ACH3
IGL3
OA
PEP−Burs
DOP2
PEP−Fmrfa3
SUB
TBA5
ACH1
TBA4
PEP−Fmrfa1
OGL2−DOP
EC
HC
TBA6
CCAP
FBL
VL
TBA3
TBA1
PEP−APWG
GLIA3
TBA7
TBA2
IGL1−OA
GLIA2
GLIA1
PCDHs
LOC115219216
LOC115219529
LOC115219542
LOC115218947
LOC115218988
LOC115219537
LOC115218437
LOC115218739
LOC118765869
LOC115218743
LOC115218977
LOC115219480
LOC115218757
LOC115219543
LOC115218987
LOC115219232
LOC115218975
LOC115218949
LOC115221427
LOC115218938
LOC115219368
LOC115218950
LOC115218983
LOC115219115
LOC115219527
LOC115218737
LOC115219405
LOC115219365
LOC115219183
LOC115219545
LOC115218862
LOC115218755
LOC115218986
LOC115218763
LOC115218740
LOC115219116
LOC115229210
LOC115230600
LOC115218736
LOC115218969
LOC115219377
LOC115219532
LOC118766074
LOC115218744
LOC115219535
LOC115219544
LOC115219231
LOC115218750
LOC118766058
LOC115219528
LOC115219541
LOC115218965
LOC115218746
LOC115219233
LOC115218747
LOC115218738
LOC115219540
LOC115219531
LOC115218953
LOC115219474
LOC115219237
LOC115219489
LOC115219236
LOC115218982
LOC115218974
LOC115218981
LOC115218741
LOC115218758
Avg Exp
6
4
2
0
−2
b
Cell types
PREC
HC
GLUT4
FBL
PEP−Fmrfa3
TBA8
OA
IGL3
SUB
ACH1
GLUT3
IGL2−GLUT/DOP
GLUT1
PEP−Fmrfa1
TBA3
CCAP
ACH3
PEP−Burs
GABA
SERT
DOP1
DOP2
IGL1−OA
ACH2
GLIA3
DOP3
IGL4−L11
TBA4
TBA7
TBA6
GLUT2
TBA5
TBA2
OGL3−OA
TBA1
PEP−APWG
OGL2−DOP
OGL1
VL
GLIA1
GLIA2
EC
ZnFs
Avg Exp
6
4
2
0
−2

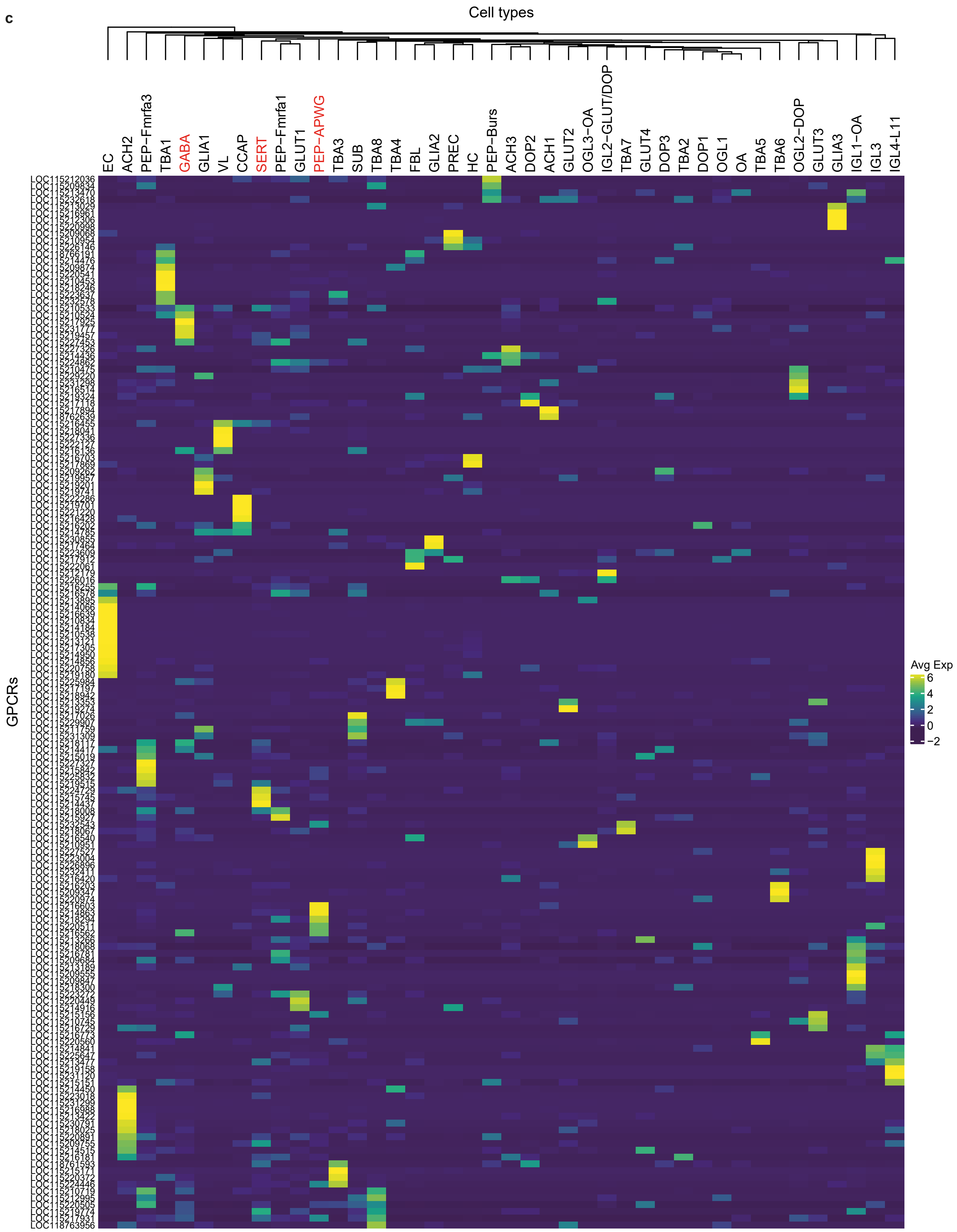

Supplementary Fig. 21 Average expression of octopus specific genes per cell type. Scaled heatmap of differentially expressed protocadherins in **a**, Zinc-finger transcription factors in **b**, and GPCR-coupled receptors in **c**, averaged per cell type (pseudo-bulk). Significant enrichments are highlighted in red.

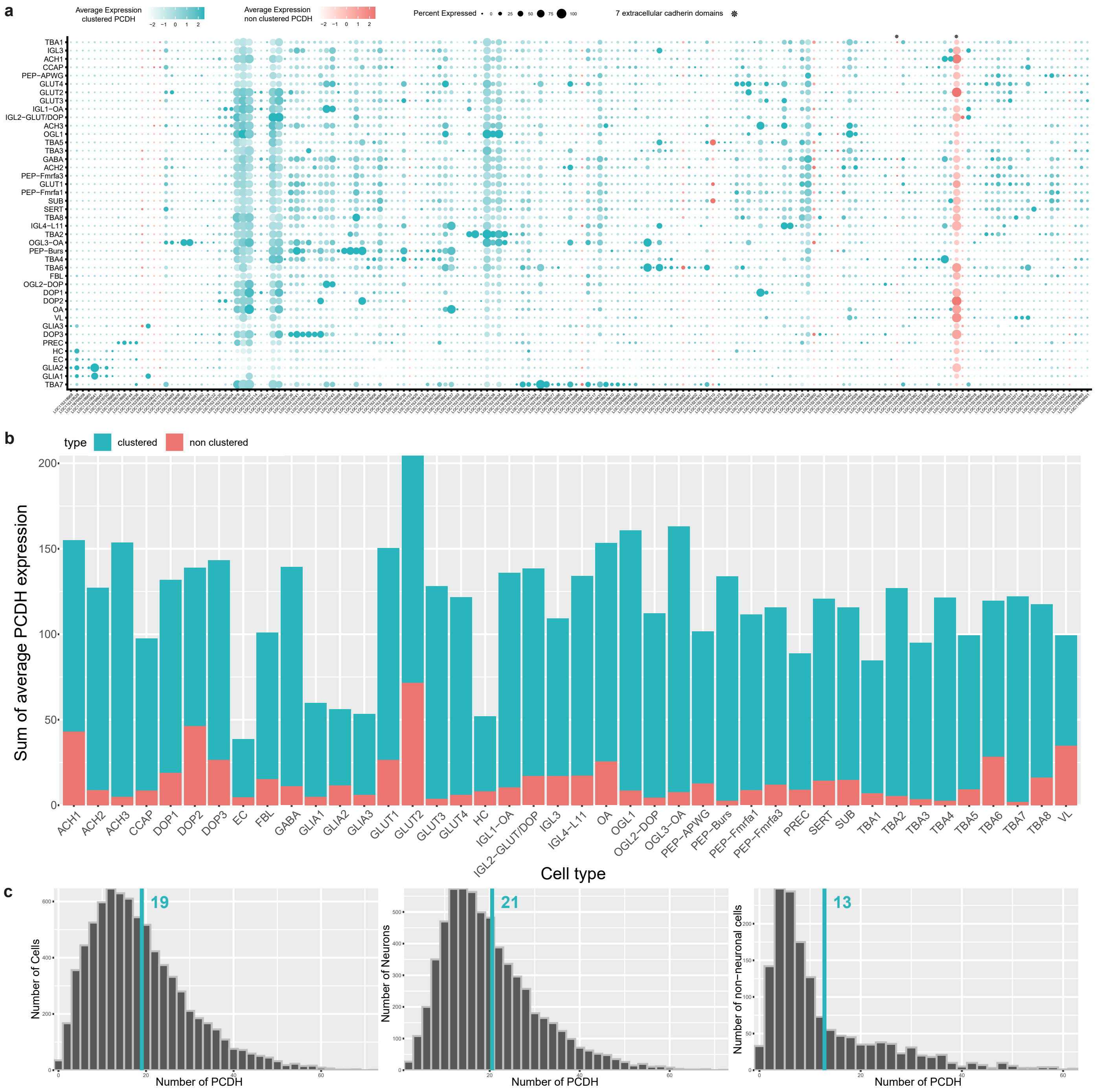

Supplementary Fig. 22 Single-cell and cell-type level expression of all 172 PCDH detected. **a** Dot plot visualization of the expression of all PCDH per cell type. **b** Stacked bar plot indicates the total average expression of all PCDHs per cell type (sum of average expression across all PCDHs). **c** Frequency distribution of the number of different PCDH expressed per cell (raw counts > 0), neurons and non-neuronal cells. Average is indicated by the intercept. Total number of cells analysed; 7726, of which 6212 were neurons and 1514 were non-neuronal. PCDH type is color-coded in a,b; 159 clustered PCDH and 13 non-clustered PCDH were detected.

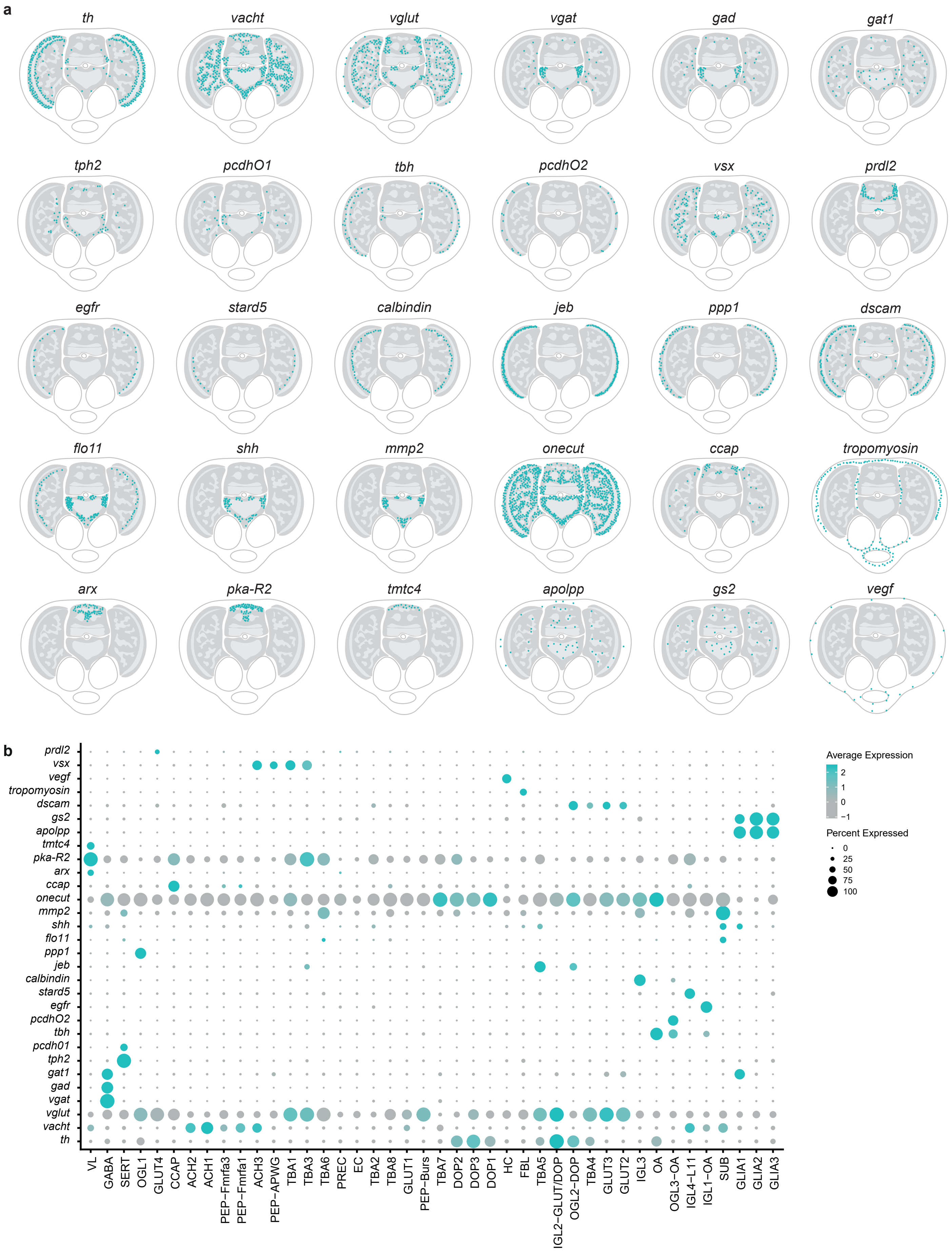

Supplementary Fig. 23 Schematic overview of cell type markers. **a** Scheme of all *in situ* hybridizations shown in this study (signal shown in blue). **b** Dot plot of all marker genes.

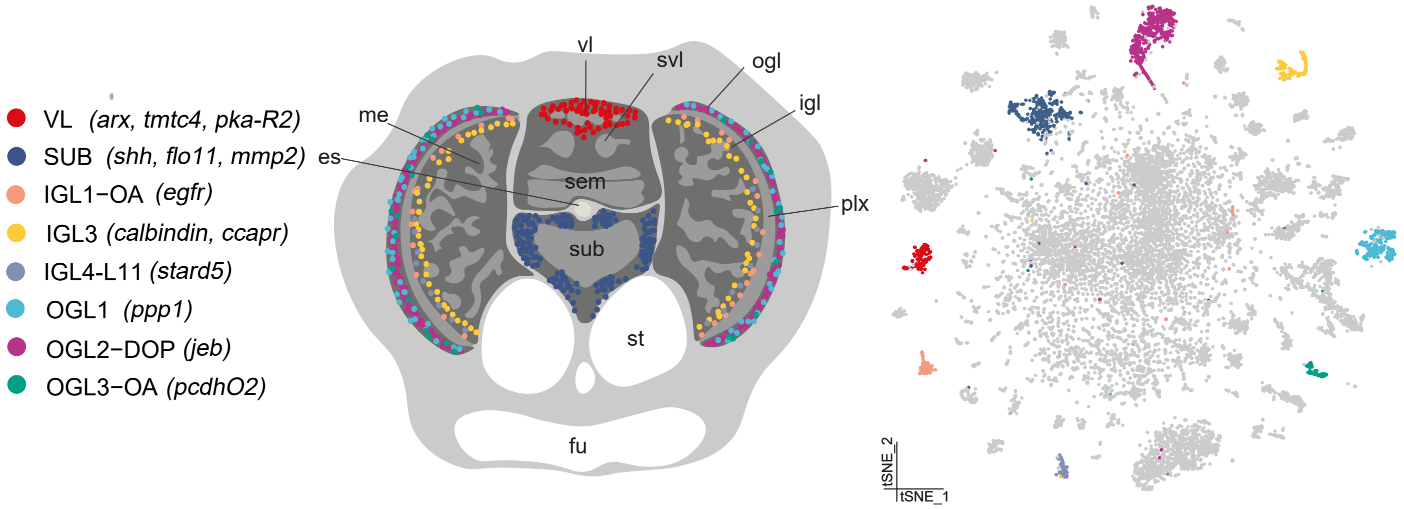

Supplementary Fig. 24 Schematic overview of cell types that are spatially confined. Scheme illustrates the spatial distribution of the different cell types within the brain and within the t-SNE plot. Cell types are color coded and marker genes are visualized between brackets. DOP, dopaminergic; es, esophagus; fu, funnel; igl, inner granular layer; me, medulla; OA, octopaminergic; ogl, outer granular layer; plx, plexiform layer; sem, supra-esophageal mass; st, statocysts; sub, sub-esophageal mass; svl, subvertical lobe; vl, vertical lobe.
