# Supplementary material for: Cell type diversity in a developing octopus brain
Source: Nat Commun. 2022 Nov 30;13:7392. doi: 10.1038/s41467-022-35198-1 (PMC9712504; doi:10.1038/s41467-022-35198-1)
Supplement: Supplementary file 5 — Reporting Summary [file 41467_2022_35198_MOESM5_ESM.pdf]

nature portfolio

Corresponding author(s): Eve Seuntjens

Last updated by author(s): Nov 14, 2022

Reporting Summary

Nature Portfolio wishes to improve the reproducibility of the work that we publish. This form provides structure for consistency and transparency in reporting. For further information on Nature Portfolio policies, see our Editorial Policies and the Editorial Policy Checklist.

Statistics

For all statistical analyses, confirm that the following items are present in the figure legend, table legend, main text, or Methods section.

n/a	Confirmed	
☐	☒	The exact sample size (*n*) for each experimental group/condition, given as a discrete number and unit of measurement
☐	☒	A statement on whether measurements were taken from distinct samples or whether the same sample was measured repeatedly
☐	☒	The statistical test(s) used AND whether they are one- or two-sided<br>*Only common tests should be described solely by name; describe more complex techniques in the Methods section.*
☒	☐	A description of all covariates tested
☐	☒	A description of any assumptions or corrections, such as tests of normality and adjustment for multiple comparisons
☒	☐	A full description of the statistical parameters including central tendency (e.g. means) or other basic estimates (e.g. regression coefficient) AND variation (e.g. standard deviation) or associated estimates of uncertainty (e.g. confidence intervals)
☐	☒	For null hypothesis testing, the test statistic (e.g. *F*, *t*, *r*) with confidence intervals, effect sizes, degrees of freedom and *P* value noted<br>*Give P values as exact values whenever suitable.*
☒	☐	For Bayesian analysis, information on the choice of priors and Markov chain Monte Carlo settings
☒	☐	For hierarchical and complex designs, identification of the appropriate level for tests and full reporting of outcomes
☒	☐	Estimates of effect sizes (e.g. Cohen's *d*, Pearson's *r*), indicating how they were calculated

Our web collection on statistics for biologists contains articles on many of the points above.

Software and code

Policy information about availability of computer code

Data collection	No software was used to collect the data.
Data analysis	Commercial and open source code:<br>TransDecoder v5.5.0, BLAST+ v2.7.1, EggNOG-mapper v2, GSEApy v0.10.3, insitu_probe_generator v0.3.2, 10x Genomics Cell Ranger v5.0.1, Seurat v3.2.3 for initial data analysis, Seurat v4.0.4 for data visualization, scclusteval v1.0, SCopeLoomR v.0.13.0, PrctCellExpringGene function (From Github/Ryan-Zhu, https://github.com/satijalab/seurat/issues/371), ComplexHeatmap v2.8.0, Tspex v0.6.2, SAMap v0.1.6, iCytoTRACE, DESeq2 v1.36.0, Possvm, Ape v5.6-2, R v4.0.1-4.2.2, iTOL v6<br>Custom tool used: https://github.com/SeuntjensLab/easy_hcr<br>All the R and python scripts that were used for genome annotation and data analysis is also available on GitHub; https://github.com/SeuntjensLab/Styfhals_2022 and https://github.com/rajewsky-lab/octopus_microRNAs/tree/main/gene_extension.<br>An archive of the GitHub repository can be found here; https://zenodo.org/record/7317825.

For manuscripts utilizing custom algorithms or software that are central to the research but not yet described in published literature, software must be made available to editors and reviewers. We strongly encourage code deposition in a community repository (e.g. GitHub). See the Nature Portfolio guidelines for submitting code & software for further information.

Data

Policy information about availability of data

All manuscripts must include a data availability statement. This statement should provide the following information, where applicable:

- Accession codes, unique identifiers, or web links for publicly available datasets
- A description of any restrictions on data availability
- For clinical datasets or third party data, please ensure that the statement adheres to our policy

The scRNA-seq and snRNA-seq data generated in this study have been deposited in the Gene Expression Omnibus database under accession code GSE193622 [https://www.ncbi.nlm.nih.gov/geo/query/acc.cgi?acc=GSE193622]. Analyzed and integrated single cell and nuclei data are available online at https://scope.aertslab.org/#/Octopus_Brain/. SCope allows for easy simultaneous visualization of the expression of three genes while toggling between different embeddings. Marker gene lists can also be downloaded here for different clusterings :
- Seurat clustering [https://scope.aertslab.org/#/Octopus_Brain/Octopus_Brain%2Fparalarval_brain_seurat.loom/gene]
- Annotated clustering [https://scope.aertslab.org/#/Octopus_Brain/Octopus_Brain%2Fparalarval_brain_annotated.loom/gene].

Other datasets used in this study can be found here;
- Genome assembly for Octopus sinensis; ASM634580v1 [https://www.ncbi.nlm.nih.gov/data-hub/genome/GCF_006345805.1/]
- Embryonic and paralarval Iso-Seq (PacBio Sequel) data for Octopus vulgaris; PRJNA718058 [https://www.ncbi.nlm.nih.gov/bioproject/?term=PRJNA718058]
- Adult Iso-Seq and FLAM-Seq data for Octopus vulgaris; PRJNA791920 [https://www.ncbi.nlm.nih.gov/bioproject/?term=PRJNA791920]
- Paralarval bulk RNAseq data for Octopus vulgaris; PRJNA547720 [https://www.ncbi.nlm.nih.gov/bioproject/?term=PRJNA547720]
- Adult bulk RNAseq data for Octopus vulgaris; ArrayExpress database under accession number E-MTAB-3957 [https://www.ebi.ac.uk/biostudies/arrayexpress/studies/E-MTAB-3957?query=E-MTAB-3957]

Human research participants

Policy information about studies involving human research participants and Sex and Gender in Research.

Reporting on sex and gender	NA
Population characteristics	NA
Recruitment	NA
Ethics oversight	NA

Note that full information on the approval of the study protocol must also be provided in the manuscript.

Field-specific reporting

Please select the one below that is the best fit for your research. If you are not sure, read the appropriate sections before making your selection.

☒ Life sciences ☐ Behavioural & social sciences ☐ Ecological, evolutionary & environmental sciences

For a reference copy of the document with all sections, see nature.com/documents/nr-reporting-summary-flat.pdf

Life sciences study design

All studies must disclose on these points even when the disclosure is negative.

Sample size	No sample-size calculation was performed. Based on the total number of cells present in the brain (200,000) and on previous dissociation experiments, we estimated that 30 brains per sample would ensure sufficient number of cells and nuclei.
Data exclusions	We filtered the dataset following Seurat's recommendations to ensure high quality cells/nuclei. Nuclei and cells with too high (>4000) or too low (<400 for nuclei, <800 for cells) gene counts were filtered out. Cells with a higher percentage (>5) of mitochondrial RNA were regressed out. Genes expressed in less than 10 cells were excluded.
Replication	Our dataset contains data points of two samples; cells and nuclei. We identified similar cell types in both samples even though the sequencing method was different, which provides strong evidence that these are meaningful biological cell types. Moreover, in our analysis we focused on stable clusters, which consist out of cells that consistently cluster together independent of clustering parameters. Small differences existed between the nuclei-cells, where some cell types were less abundant in the nuclei (ACH1, ACH3, GLIA3, Pep-burs) while others were underrepresented in the cells (FBL, GLIA2).
Randomization	No randomization strategies were applied. We pooled brains from animals that were a similar age and originated from the same mother.

Blinding	Blinding is not relevant to this exploratory study.

Reporting for specific materials, systems and methods

We require information from authors about some types of materials, experimental systems and methods used in many studies. Here, indicate whether each material, system or method listed is relevant to your study. If you are not sure if a list item applies to your research, read the appropriate section before selecting a response.

Materials & experimental systems

n/a	Involved in the study
☐	☒ Antibodies
☒	☐ Eukaryotic cell lines
☒	☐ Palaeontology and archaeology
☐	☒ Animals and other organisms
☒	☐ Clinical data
☒	☐ Dual use research of concern

Methods

n/a	Involved in the study
☒	☐ ChIP-seq
☒	☐ Flow cytometry
☒	☐ MRI-based neuroimaging

Antibodies

Antibodies used	Anti-Tubulin, Acetylated antibody (Mouse Monoclonal), Sigma, T6793, clone 6-11B-1, BATCH 0000108923, dilution used 1:300<br>Anti-phospho-Histone H3 (Ser10) antibody (Rabbit Polyclonal), Millipore, 06-570, LOT3527703, dilution used 1:300<br>Donkey anti-Mouse IgG (H+L) Secondary Antibody, Alexa Fluor 488, Life Tech (Invitrogen), A-21202, LOT2266877, dilution used 1:300<br>Donkey anti-Rabbit IgG (H+L) Highly Cross-Adsorbed Secondary Antibody, Alexa Fluor 555, Life Tech (Invitrogen), A-31572, LOT2286312, dilution used 1:300
Validation	These antibodies have been used in octopus in previous studies (Deryckere et al., 2021).

Animals and other research organisms

Policy information about studies involving animals; ARRIVE guidelines recommended for reporting animal research, and Sex and Gender in Research

Laboratory animals	O. vulgaris embryos were obtained from the Instituto Español de Oceanografía (IEO, Tenerife, Spain). Embryos were then incubated until hatching in a closed system in the Laboratory of Developmental Neurobiology (KU Leuven, Belgium). One day old paralarvae were used for all experiments in this study.
Wild animals	Adult O.vulgaris females were captured from the wild by fishermen in Tenerife, Spain. These animals were housed in the Instituto Español de Oceanografía where they spawned naturally. Embryos were subsequently taken from the mother and express shipped to Belgium. After spawning, the mother undergoes a natural death.
Reporting on sex	Sex was unknown.
Field-collected samples	The study did not involve samples collected from the field.
Ethics oversight	All procedures involving hatchlings were approved by the ethical board on animal experimentation from KU Leuven (permit P080/2021), in compliance with the Directive 2010/63/EU.

Note that full information on the approval of the study protocol must also be provided in the manuscript.
